# Supplementary material for: Early warning score adjusted for age to predict the composite outcome of mortality, cardiac arrest or unplanned intensive care unit admission using observational vital-sign data: a multicentre development and validation
Source: BMJ Open. 2019 Nov 19;9(11):e033301. doi: 10.1136/bmjopen-2019-033301 (PMC6887005; doi:10.1136/bmjopen-2019-033301)
Supplement: Supplementary data [file bmjopen-2019-033301supp001.pdf]

Appendix A

Figure A1. The receiver operating characteristics (ROC) curve evaluated on the two validation sets, Oxford University Hospitals NHS Trust denoted as 'OUH validation set' and the Portsmouth NHS Trust validation set, for the Age-specific Early Warning Score (ASEWS), Manual Centile-based Early Warning (MCEWS), and the National Early Warning Score (NEWS), for the combined outcome of mortality, cardiac arrest or unplanned ICU admission within 24 hours of vital signs observations.

OUH Validation Set

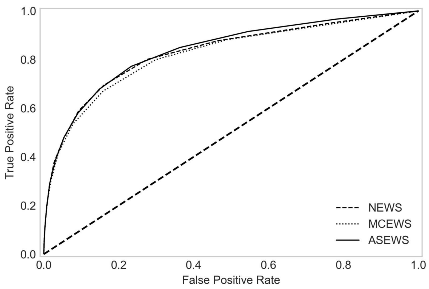

PH Validation Set

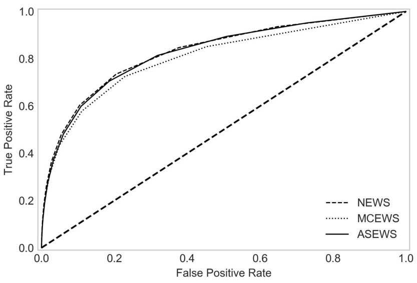

16 – 45 year olds

OUH Validation Set

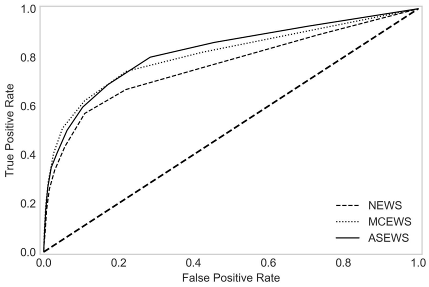

PH Validation Set

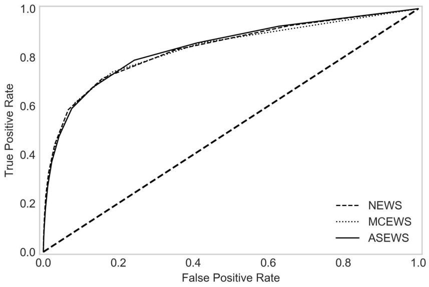

>= 46 year olds

OUH Validation Set

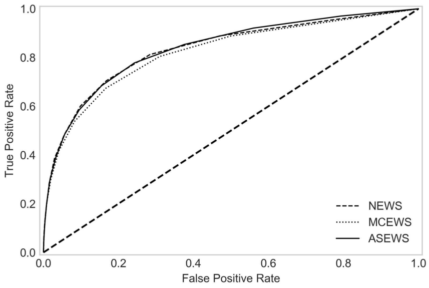

PH Validation Set

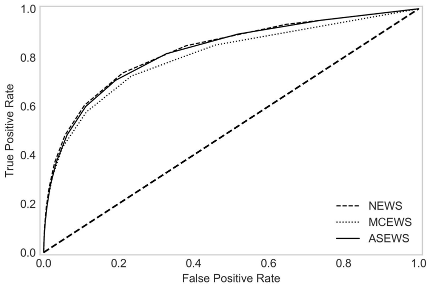

Figure A2: Efficiency EWS curves plotting sensitivity (%) (x-axis) against the percentage of observations with an EWS score greater than or equal to a given EWS threshold, denoted as positives on the y-axis, for the two best performing models, the Age-Specific Early Warning Score (ASEWS) and the National Early Warning Score (NEWS) across the overall population, 16-45 years old, and older than 45 years old in the OUH and PH validation sets. The blue lines compare the trigger rates of the EWS systems at a fixed sensitivity of 80%. The most notable difference is for the 16-45 year olds, where ASEWS produces less triggers than NEWS to achieve the same sensitivity of 80%.

(a) Overall patient population

OUH Validation Set

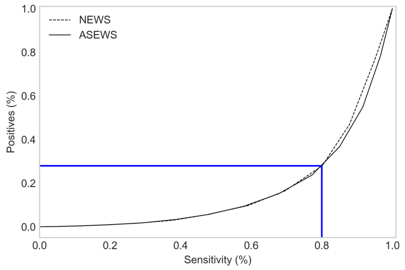

Both ASEWS and NEWS have a similar trigger rate of ~ 27.9%.

PH Validation Set

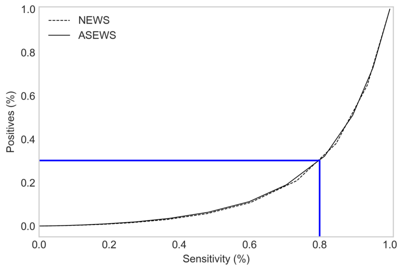

Both ASEWS and NEWS have a similar trigger rate of ~ 30.2%.

(b) 16 – 45 years old

OUH Validation Set

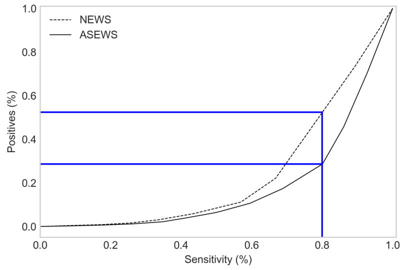

ASEWS has a trigger rate of ~ 28.6% while NEWS has a higher trigger rate of ~52.4%.

PH Validation Set

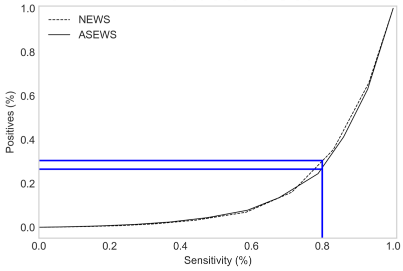

ASEWS has a trigger rate of ~ 26.5% while NEWS has a higher trigger rate of ~30.4%.

(c) >= 46 years old

OUH Validation Set

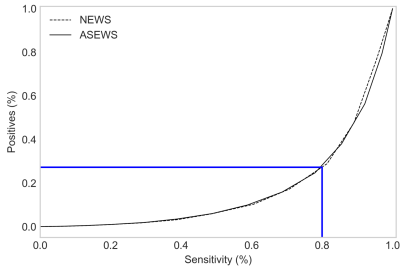

Both ASEWS and NEWS have a similar trigger rate of ~ 27.2%.

PH Validation Set

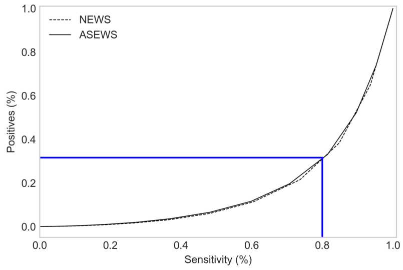

Both ASEWS and NEWS have a similar trigger rate of ~ 31.5%.

Table A1

(1) OUH Hospitals

Hospital	Size (hectares)	Catchment area	Number of beds	Percentage of attendance in 2013/2014[1]
John Radcliffe Hospital[2]	26.7	Oxfordshire	832	42.2%
Horton General Hospital[3]	9.9	Northern Oxfordshire and neighbouring communities in south Northamptonshire and south-east Warwickshire.[4]	248	11.2%
Nuffield Orthopaedic Centre[5]	8.9	Oxfordshire	160	13.4%
Churchill Hospital[6]	28.3	Oxfordshire	217	23.7%

(2) PH Hospital

Hospital	Catchment area	Number of beds	Total attendances (emergency/elective)
Queen Alexandria Hospital[7]	Portsmouth and surrounding areas of South East Hampshire	1,250	566,000

[1] https://www.ouh.nhs.uk/about/publications/documents/2.TrustProfile.pdf
[2] https://www.cqc.org.uk/location/RTH08/reports
[3] https://www.cqc.org.uk/location/RTH05/reports
[4] https://www.ouh.nhs.uk/about/publications/documents/4.MarketAssessment.pdf
[5] https://www.cqc.org.uk/location/RTH03/reports
[6] https://www.cqc.org.uk/sites/default/files/new_reports/AAAA0627.pdf
[7] https://www.cqc.org.uk/provider/RHU/reports

Table A2: The number of True positives (TP), False Negatives (FN), False Positives (FP), True Negatives (TN) and Positive Predictive Values (PPV) at each possible total score for the Age-specific Early Warning Score (ASEWS) and the National Early Warning Score (NEWS) as validated on the (1) OUH validation set and the (2) Portsmouth validation set for the (a) overall population, (b) 16 – 45 years old, and (c) >= 46 years old.

1. **OUH Validation Set**
   a. **Overall population**

Total EWS score	ASEWS					NEWS				
	TP	FN	FP	TN	PPV	TP	FN	FP	TN	PPV
0	3776	0	577795	0	0.01	3776	0	577795	0	0.01
1	3649	127	451477	126318	0.01	3586	190	441445	136350	0.01
2	3457	319	315372	262423	0.01	3314	462	268972	308823	0.01
3	3208	568	209897	367898	0.02	3023	753	159369	418426	0.02
4	2915	861	134765	443030	0.02	2623	1153	92603	485192	0.03
5	2561	1215	85605	492190	0.03	2225	1551	53664	524131	0.04
6	2189	1587	52549	525246	0.04	1787	1989	30078	547717	0.06
7	1814	1962	31087	546708	0.06	1422	2354	15711	562084	0.08
8	1455	2321	17725	560070	0.08	1043	2733	8131	569664	0.11
9	1107	2669	9267	568528	0.11	670	3106	3876	573919	0.15
10	751	3025	4546	573249	0.14	444	3332	1761	576034	0.2
11	470	3306	2061	575734	0.19	296	3480	811	576984	0.27
12	276	3500	870	576925	0.24	194	3582	346	577449	0.36
13	165	3611	332	577463	0.33	102	3674	139	577656	0.42
14	88	3688	130	577665	0.4	55	3721	58	577737	0.49
15	42	3734	43	577752	0.49	29	3747	14	577781	0.67
16	25	3751	12	577783	0.68	18	3758	7	577788	0.72
17	18	3758	8	577787	0.69	8	3768	1	577794	0.89
18	9	3767	3	577792	0.75	4	3772	0	577795	1.0
19	6	3770	1	577794	0.86	0	3776	0	577795	-
20	4	3772	0	577795	1.0	0	3776	0	577795	-

   b. **16-45 years old**

Total EWS score	ASEWS					NEWS				
	TP	FN	FP	TN	PPV	TP	FN	FP	TN	PPV
0	346	0	79161	0	0.0	346	0	79161	0	0.0
1	321	25	55742	23419	0.01	309	37	58067	21094	0.01
2	298	48	36011	43150	0.01	262	84	33461	45700	0.01
3	277	69	22488	56673	0.01	231	115	17359	61802	0.01
4	238	108	13548	65613	0.02	197	149	8655	70506	0.02
5	206	140	8222	70939	0.02	150	196	4464	74697	0.03
6	173	173	4914	74247	0.03	116	230	2286	76875	0.05
7	141	205	2820	76341	0.05	89	257	1159	78002	0.07
8	120	226	1568	77593	0.07	59	287	578	78583	0.09
9	91	255	825	78336	0.1	30	316	302	78859	0.09

10	60	286	415	78746	0.13	15	331	115	79046	0.12
11	31	315	170	78991	0.15	9	337	34	79127	0.21
12	15	331	69	79092	0.18	7	339	14	79147	0.33
13	9	337	25	79136	0.26	4	342	5	79156	0.44
14	4	342	13	79148	0.24	1	345	1	79160	0.5
15	2	344	4	79157	0.33	1	345	0	79161	1.0
16	0	346	1	79160	0.0	0	346	0	79161	-
17	0	346	0	79161	-	0	346	0	79161	-
18	0	346	0	79161	-	0	346	0	79161	-
19	0	346	0	79161	-	0	346	0	79161	-
20	0	346	0	79161	-	0	346	0	79161	-

c. >= 46 years old

Total EWS score	ASEWS					NEWS				
	**TP**	**FN**	**FP**	**TN**	**PPV**	**TP**	**FN**	**FP**	**TN**	**PPV**
0	3430	0	498634	0	0.01	3430	0	498634	0	0.01
1	3328	102	395735	102899	0.01	3277	153	383378	115256	0.01
2	3159	271	279361	219273	0.01	3052	378	235511	263123	0.01
3	2931	499	187409	311225	0.02	2792	638	142010	356624	0.02
4	2677	753	121217	377417	0.02	2426	1004	83948	414686	0.03
5	2355	1075	77383	421251	0.03	2075	1355	49200	449434	0.04
6	2016	1414	47635	450999	0.04	1671	1759	27792	470842	0.06
7	1673	1757	28267	470367	0.06	1333	2097	14552	484082	0.08
8	1335	2095	16157	482477	0.08	984	2446	7553	491081	0.12
9	1016	2414	8442	490192	0.11	640	2790	3574	495060	0.15
10	691	2739	4131	494503	0.14	429	3001	1646	496988	0.21
11	439	2991	1891	496743	0.19	287	3143	777	497857	0.27
12	261	3169	801	497833	0.25	187	3243	332	498302	0.36
13	156	3274	307	498327	0.34	98	3332	134	498500	0.42
14	84	3346	117	498517	0.42	54	3376	57	498577	0.49
15	40	3390	39	498595	0.51	28	3402	14	498620	0.67
16	25	3405	11	498623	0.69	18	3412	7	498627	0.72
17	18	3412	8	498626	0.69	8	3422	1	498633	0.89
18	9	3421	3	498631	0.75	4	3426	0	498634	1.0
19	6	3424	1	498633	0.86	0	3430	0	498634	-
20	4	3426	0	498634	1.0	0	3430	0	498634	-

2. **PH Validation Set**

a. **Overall population**

Total EWS score	ASEWS TP	ASEWS FN	ASEWS FP	ASEWS TN	ASEWS PPV	NEWS TP	NEWS FN	NEWS FP	NEWS TN	NEWS PPV
0	43688	0	5822309	0	0.01	43688	0	5822309	0	0.01
1	41574	2114	4239807	1582502	0.01	40884	2804	3771232	2051077	0.01
2	39067	4621	2952112	2870197	0.01	37012	6676	2193657	3628652	0.02
3	35557	8131	1852090	3970219	0.02	32127	11561	1194727	4627582	0.03
4	30810	12878	1082260	4740049	0.03	26358	17330	608462	5213847	0.04
5	26161	17527	631288	5191021	0.04	20951	22737	315354	5506955	0.06
6	21050	22638	350149	5472160	0.06	15961	27727	158894	5663415	0.09
7	16206	27482	188860	5633449	0.08	11441	32247	77263	5745046	0.13
8	11979	31709	98384	5723925	0.11	7960	35728	37033	5785276	0.18
9	8157	35531	47771	5774538	0.15	5115	38573	16233	5806076	0.24
10	5326	38362	21912	5800397	0.2	3170	40518	6838	5815471	0.32
11	3274	40414	9204	5813105	0.26	1874	41814	2682	5819627	0.41
12	1856	41832	3391	5818918	0.35	1027	42661	1040	5821269	0.5
13	995	42693	1140	5821169	0.47	515	43173	384	5821925	0.57
14	529	43159	396	5821913	0.57	239	43449	125	5822184	0.66
15	241	43447	125	5822184	0.66	92	43596	42	5822267	0.69
16	116	43572	39	5822270	0.75	33	43655	14	5822295	0.7
17	56	43632	10	5822299	0.85	11	43677	6	5822303	0.65
18	14	43674	2	5822307	0.88	3	43685	0	5822309	1.0
19	10	43678	0	5822309	1.0	1	43687	0	5822309	1.0
20	4	43684	0	5822309	1.0	1	43687	0	5822309	1.0

b. **16 – 45 years old**

Total EWS score	ASEWS TP	ASEWS FN	ASEWS FP	ASEWS TN	ASEWS PPV	NEWS TP	NEWS FN	NEWS FP	NEWS TN	NEWS PPV
0	2399	0	602429	0	0.0	2399	0	602429	0	0.0
1	2227	172	378845	223584	0.01	2233	166	396178	206251	0.01
2	2062	337	247192	355237	0.01	1993	406	212273	390156	0.01
3	1891	508	146065	456364	0.01	1712	687	95038	507391	0.02
4	1626	773	80018	522411	0.02	1403	996	39994	562435	0.03
5	1414	985	45531	556898	0.03	1060	1339	18217	584212	0.05
6	1140	1259	25143	577286	0.04	784	1615	8428	594001	0.09
7	888	1511	13450	588979	0.06	554	1845	3844	598585	0.13
8	649	1750	6997	595432	0.08	361	2038	1799	600630	0.17
9	426	1973	3339	599090	0.11	221	2178	796	601633	0.22
10	254	2145	1491	600938	0.15	146	2253	352	602077	0.29
11	147	2252	612	601817	0.19	81	2318	139	602290	0.37
12	69	2330	235	602194	0.23	39	2360	51	602378	0.43
13	38	2361	73	602356	0.34	18	2381	18	602411	0.5
14	19	2380	22	602407	0.46	8	2391	6	602423	0.57

15	13	2386	10	602419	0.57	1	2398	2	602427	0.33
16	4	2395	2	602427	0.67	0	2399	0	602429	-
17	2	2397	1	602428	0.67	0	2399	0	602429	-
18	0	2399	0	602429	-	0	2399	0	602429	-
19	0	2399	0	602429	-	0	2399	0	602429	-
20	0	2399	0	602429	-	0	2399	0	602429	-

c. >= 46 years old

Total EWS score	ASEWS					NEWS				
	**TP**	**FN**	**FP**	**TN**	**PPV**	**TP**	**FN**	**FP**	**TN**	**PPV**
0	41289	0	5219880	0	0.01	41289	0	5219880	0	0.01
1	39347	1942	3860962	1358918	0.01	38651	2638	3375054	1844826	0.01
2	37005	4284	2704920	2514960	0.01	35019	6270	1981384	3238496	0.02
3	33666	7623	1706025	3513855	0.02	30415	10874	1099689	4120191	0.03
4	29184	12105	1002242	4217638	0.03	24955	16334	568468	4651412	0.04
5	24747	16542	585757	4634123	0.04	19891	21398	297137	4922743	0.06
6	19910	21379	325006	4894874	0.06	15177	26112	150466	5069414	0.09
7	15318	25971	175410	5044470	0.08	10887	30402	73419	5146461	0.13
8	11330	29959	91387	5128493	0.11	7599	33690	35234	5184646	0.18
9	7731	33558	44432	5175448	0.15	4894	36395	15437	5204443	0.24
10	5072	36217	20421	5199459	0.2	3024	38265	6486	5213394	0.32
11	3127	38162	8592	5211288	0.27	1793	39496	2543	5217337	0.41
12	1787	39502	3156	5216724	0.36	988	40301	989	5218891	0.5
13	957	40332	1067	5218813	0.47	497	40792	366	5219514	0.58
14	510	40779	374	5219506	0.58	231	41058	119	5219761	0.66
15	228	41061	115	5219765	0.66	91	41198	40	5219840	0.69
16	112	41177	37	5219843	0.75	33	41256	14	5219866	0.7
17	54	41235	9	5219871	0.86	11	41278	6	5219874	0.65
18	14	41275	2	5219878	0.88	3	41286	0	5219880	1.0
19	10	41279	0	5219880	1.0	1	41288	0	5219880	1.0
20	4	41285	0	5219880	1.0	1	41288	0	5219880	1.0
