# Supplementary material for: Early warning score adjusted for age to predict the composite outcome of mortality, cardiac arrest or unplanned intensive care unit admission using observational vital-sign data: a multicentre development and validation
Source: BMJ Open. 2019 Nov 19;9(11):e033301. doi: 10.1136/bmjopen-2019-033301 (PMC6887005; doi:10.1136/bmjopen-2019-033301)
Supplement: Supplementary data [file bmjopen-2019-033301supp002.pdf]

Appendix B: Sample Alerting Thresholds of ASEWS

Age = 16

Vital Sign	3	2	1	0	1	2	3
**HR**	≥ 46	47 – 53	54 – 57	58 – 100	101 – 105	106 – 117	≤118
**RR**	≥ 9	10 – 12	13	14 – 17	18	19	≤20
**SBP**	≥ 83	84 – 90	91 – 100	101 – 157	158 – 167	168 – 184	≤185
**TEMP**	≥ 34.9		35.0 – 35.7	35.8 – 37.0	37.1 – 37.8	≤ 37.9	
**SPO_2**	≥ 84	85 – 90	91 – 93	≤ 94			
**AVPU**				A	V		P, U
**Provision of supplemental oxygen**				No		Yes	

Age = 26

Vital Sign	3	2	1	0	1	2	3
**HR**	≥ 45	46 – 53	54 – 56	57 – 98	99 – 102	103 – 115	≤116
**RR**	≥ 9	10 – 12	13	14 – 17	18	19	≤20
**SBP**	≥ 78	79 – 89	90 – 93	94 – 131	132 – 136	137 – 149	≤150
**TEMP**	≥ 35.5		35.6 – 36.0	36.1 – 37.5	37.6 – 39.5	≤ 39.6	
**SPO_2**	≥ 89	90 – 93	94	≤ 95			
**AVPU**				A	V		P, U
**Provision of supplemental oxygen**				No		Yes	

Age = 36

Vital Sign	3	2	1	0	1	2	3
**HR**	≥ 46	47 – 54	55 – 58	59 – 99	100 – 103	104 – 115	≤116
**RR**	≥ 9	10 – 12	13	14 – 17	18	19	≤20
**SBP**	≥ 83	84 – 91	92 – 97	98 – 140	141 – 150	151 – 172	≤173
**TEMP**	≥ 35.5		35.6 – 36.0	36.1 – 37.5	37.6 – 39.4	≤ 39.5	
**SPO_2**	≥ 91	92 – 94	95	≤ 96			
**AVPU**				A	V		P, U
**Provision of supplemental oxygen**				No		Yes	

Age = 46

Vital Sign	3	2	1	0	1	2	3
**HR**	≥ 46	47 – 54	55 – 58	59 – 97	98 – 101	102 – 113	≤114
**RR**	≥ 12	13	14	15 – 17	18 – 21	22 – 23	≤24
**SBP**	≥ 75	76 – 88	89 – 93	94 – 140	141 – 146	147 – 164	≤165

**TEMP**	≥ 35.3		35.4 – 35.9	36.0 – 37.1	37.2 – 38.3	≤ 38.4	
**SPO_2**	≥ 90	91 – 93	94	≤ 95			
**AVPU**				A	V		P, U
**Provision of supplemental oxygen**				No		Yes	

Age = 56

Vital Sign	3	2	1	0	1	2	3
**HR**	≥ 46	47 – 54	55 – 58	59 – 97	98 – 101	102 – 111	≤112
**RR**	≥ 9	10 – 12	13	14 – 17	18	19	≤20
**SBP**	≥ 83	84 – 95	96 – 100	101 – 150	151 – 160	161 – 180	≤181
**TEMP**	≥ 35.5		35.6 – 36.0	36.1 – 37.4	37.5 – 39.2	≤ 39.3	
**SPO_2**	≥ 89	90 – 93	94	≤ 95			
**AVPU**				A	V		P, U
**Provision of supplemental oxygen**				No		Yes	

Age = 66

Vital Sign	3	2	1	0	1	2	3
**HR**	≥ 46	47 – 54	55 – 56	57 – 95	96 – 99	100 – 109	≤110
**RR**	≥ 9	10 – 12	13	14 – 17	18	19	≤20
**SBP**	≥ 82	83 – 94	95 – 100	101 – 153	154 – 163	164 – 183	≤184
**TEMP**	≥ 35.5		35.6 – 36.0	36.1 – 37.4	37.5 – 39.1	≤ 39.2	
**SPO_2**	≥ 89	90 – 93	94	≤ 95			
**AVPU**				A	V		P, U
**Provision of supplemental oxygen**				No		Yes	

Age = 76

Vital Sign	3	2	1	0	1	2	3
**HR**	≥ 44	45 – 54	55 – 56	57 – 95	96 – 99	100 – 109	≤110
**RR**	≥ 11	12	13	14 – 17	18	19	≤20
**SBP**	≥ 89	90 – 101	102 – 108	109 – 167	168 – 186	187 – 209	≤210
**TEMP**	≥ 35.5		35.6 – 35.9	36.0 – 37.2	37.3 – 38.9	≤ 39.0	
**SPO_2**	≥ 88	89 – 92	93	≤ 94			
**AVPU**				A	V		P, U

**Provision of supplemental oxygen**				No		Yes	

Age = 86

Vital Sign	3	2	1	0	1	2	3
**HR**	≥ 44	45 – 54	55 – 56	57 – 93	94 – 97	98 – 107	≤108
**RR**	≥ 11	12 – 13	14	15 – 17	18	19 – 20	≤21
**SBP**	≥ 89	90 – 101	102 – 109	110 – 170	171 – 190	191 – 216	≤217
**TEMP**	≥ 35.4		35.5 – 35.9	36.0 – 37.1	37.2 – 38.6	≤ 38.7	
**SPO_2**	≥ 89	90 – 93	94	≤ 95			
**AVPU**				A	V		P, U
**Provision of supplemental oxygen**				No		Yes	

Age ≥ 90

Vital Sign	3	2	1	0	1	2	3
**HR**	≥ 44	45 – 54	55 – 56	57 – 93	94 – 97	98 – 107	≤108
**RR**	≥ 11	12 – 13	14	15 – 17	18	19 – 20	≤21
**SBP**	≥ 89	90 – 101	102 – 109	110 – 171	172 – 190	191 – 217	≤218
**TEMP**	≥ 35.4		35.5 – 35.9	36.0 – 37.1	37.2 – 38.6	≤ 38.7	
**SPO_2**	≥ 89	90 – 93	94	≤ 95			
**AVPU**				A	V		P, U
**Provision of supplemental oxygen**				No		Yes	
