# Supplementary material for: Early warning score adjusted for age to predict the composite outcome of mortality, cardiac arrest or unplanned intensive care unit admission using observational vital-sign data: a multicentre development and validation
Source: BMJ Open. 2019 Nov 19;9(11):e033301. doi: 10.1136/bmjopen-2019-033301 (PMC6887005; doi:10.1136/bmjopen-2019-033301)
Supplement: Author's manuscript [file bmjopen-2019-033301.draft_revisions.pdf]

BMJ Open

BMJ Open is committed to open peer review. As part of this commitment we make the peer review history of every article we publish publicly available.

When an article is published we post the peer reviewers' comments and the authors' responses online. We also post the versions of the paper that were used during peer review. These are the versions that the peer review comments apply to.

The versions of the paper that follow are the versions that were submitted during the peer review process. They are not the versions of record or the final published versions. They should not be cited or distributed as the published version of this manuscript.

BMJ Open is an open access journal and the full, final, typeset and author-corrected version of record of the manuscript is available on our site with no access controls, subscription charges or pay-per-view fees (http://bmjopen.bmj.com).

If you have any questions on BMJ Open's open peer review process please email info.bmjopen@bmj.com

BMJ Open

Early warning score adjusted for age to predict the composite outcome of mortality, cardiac arrest or unplanned ICU admission using observational vital-sign data: a multi-center development and validation

Journal:	*BMJ Open*
Manuscript ID	bmjopen-2019-033301
Article Type:	Original research
Date Submitted by the Author:	01-Aug-2019
Complete List of Authors:	Shamout, Farah; University of Oxford, Institute of Biomedical Engineering;<br>Zhu, Tingting; University of Oxford, Institute of Biomedical Engineering<br>Clifton, Lei; University of Oxford, Nuffield Department of Population Health<br>Briggs, Jim; University of Portsmouth, Centre for Healthcare Modelling and Informatics<br>Prytherch, David ; University of Portsmouth, Centre for Healthcare Modelling and Informatics<br>Meredith, Paul; Portsmouth Hospitals NHS Trust, Research & Innovation Department<br>Tarassenko, Lionel; University of Oxford, Engineering Science<br>Watkinson, Peter; Oxford University Hospitals NHS Trust, Kadoorie Centre for Critical Care research and Education<br>Clifton, David; University of Oxford, Engineering Science
Keywords:	Health informatics < BIOTECHNOLOGY & BIOINFORMATICS, Quality in health care < HEALTH SERVICES ADMINISTRATION & MANAGEMENT, Adverse events < THERAPEUTICS

SCHOLARONE™
Manuscripts

Early warning score adjusted for age to predict the composite outcome of mortality, cardiac arrest or unplanned ICU admission using observational vital-sign data: a multicenter development and validation

Farah E. Shamout (BSc)
University of Oxford
Institute of Biomedical Engineering
Old Road Campus Research Building
University of Oxford
Oxford, OX3 7DQ, UK

Tingting Zhu (DPhil)
Stipendiary Junior Research Fellowship, St Hilda's College, University of Oxford
Institute of Biomedical Engineering
Old Road Campus Research Building
University of Oxford
Oxford, OX3 7DQ, UK

Lei Clifton (PhD)
Nuffield Department of Population Health
Big Data Institute (BDI) Building
Old Road Campus Research Building
University of Oxford
Oxford, OX3 7LF, UK

Jim S. Briggs (DPhil)
Centre for Healthcare Modelling and Informatics
Buckingham Building
University of Portsmouth
Portsmouth, PO1 3HE, UK

David R. Prytherch (PhD, MIEPM, CSci)
Centre for Healthcare Modelling and Informatics
Buckingham Building
University of Portsmouth
Portsmouth, PO1 3HE, UK

Paul Meredith (PhD)
Research & Innovation
Queen Alexandra Hospital
Portsmouth Hospitals NHS Trust
PO6 3LY, UK

Lionel Tarassenko (DPhil)
Fellow of the Royal Academy of Engineering, Fellow of the Academy of Medical Sciences
Institute of Biomedical Engineering
Old Road Campus Research Building
University of Oxford
Oxford, OX3 7DQ, UK

For peer review only - http://bmjopen.bmj.com/site/about/guidelines.xhtml

Peter J. Watkinson (MD)
Nuffield Department of Clinical Neurosciences
Oxford University Hospitals NHS Trust
Oxford, OX3 9DU, UK

David A. Clifton (DPhil)
Institute of Biomedical Engineering
Old Road Campus Research Building
University of Oxford
Oxford, OX3 7DQ, UK

Address correspondence to: Farah E. Shamout, Computational Health Informatics Lab, Institute of Biomedical Engineering, University of Oxford, Oxford, OX3 7DQ, United Kingdom
Tel.: +44 (0) 7397 082634, Email: farah.shamout@balliol.ox.ac.uk

Keywords: vital signs, early warning scores, age factors, quality improvement, multicenter study, patient outcome assessment

Word Count=2,278
Abstract Word Count=300
Number of references=19
Figures=2
Tables=4
Supplementary Digital Content=2

Funding Statement

The work of FES is funded by the Rhodes Trust. PW is supported by the NIHR Biomedical Research Centre, Oxford. This publication presents independent research commissioned by the Health Innovation Challenge Fund (HICF-R9-524; WT-103703/Z/14/Z), a parallel funding partnership between the Department of Health & Social Care and Wellcome Trust. The views expressed in this publication are those of the author(s) and not necessarily those of the Department of Health & Social Care or Wellcome Trust.

Acknowledgements

For dataset curation and extraction, we would like to thank Dr. Marco Pimentel and Dr. Oliver Redfern.

Author Contributions

Farah Shamout developed the analysis plan and undertook the data analysis and the writing of the paper. Lei Clifton guided the statistical analysis of the results. Jim Briggs, David Prytherch, & Paul Meredith collected the Portsmouth dataset and provided advice on its analysis. Peter Watkinson guided the analysis and made substantial improvements to the paper. Tingting Zhu, David Clifton and Lionel Tarassenko supervised the study and contributed to the data analysis plan.

Conflict of Interest

Lionel Tarassenko and Peter Watkinson co-developed the System for Electronic Notification and Documentation (SEND), for which Sensyne Health (SH) has purchased a sole license. SH has a research agreement with the University of Oxford and royalty agreements with Oxford University Hospitals NHS Trust and the University of Oxford. SH paid Lionel Tarassenko consultancy fees as a member of its Strategic Advisory Board. Peter Watkinson is the Chief Medical Officer and holds shares in the company. His department has received funding from SH. David Clifton is the Research Director at SH. David Prytherch was an employee of Portsmouth NHS Trust until July 2016 and he assisted the Royal College of Physicians of London in the analysis of data validating NEWS.

For peer review only - http://bmjopen.bmj.com/site/about/guidelines.xhtml

Abstract

Objectives

Early Warning Scores (EWS) alerting for in-hospital deterioration are commonly developed using routinely collected vital-sign data from the whole in-hospital population. As these in-hospital populations are dominated by those over the age of 45 years, resultant scores may perform less well in younger age groups. We developed and validated an Age-specific Early Warning Score (ASEWS) derived from statistical distributions of vital signs from age subsets.

Design: Observational Cohort Study

Setting: Oxford University Hospitals (OUH) July 2013 – March 2018 and Portsmouth Hospitals (PH) NHS Trust January 2010 – March 2017 within the HAVEN database

Participants: Hospitalised patients with electronically documented vital-sign observations

Outcome: Composite outcome of unplanned ICU admission, mortality, and cardiac arrest.

Methods and Results:

Statistical distributions of vital signs were used to develop an age-specific early warning score to predict the composite outcome within 24 hours. The OUH development set consisted of 2,538,099 vital-sign observation sets from 142,806 admissions (mean age (std): 59.8 (20.3)). We compared the performance of ASEWS to the National Early Warning Score (NEWS) and our previous EWS (MCEWS) on an OUH validation set consisting of 581,571 observation sets from 25,407 emergency admissions (mean age (std): 63.0 (21.4)) and a PH validation set consisting of 5,865,997 observation sets from 233,632 emergency admissions (mean age (std): 64.3 (21.1)). ASEWS performed better in the 16-45 years age group in the OUH validation set (AUROC 0.820 [95% CI 0.815-0.824]) and PH validation set (AUROC 0.840 [95% CI 0.839-0.841]) than NEWS (AUROC 0.763 [95% CI 0.758-0.768] and AUROC 0.836 [95% CI 0.835 0 0.838] respectively) and MCEWS (AUROC 0.808 [95% CI 0.803-0.812] and AUROC 0.833 [95% CI 0.831 - 0.834] respectively). Differences in performance were not consistent in the elder age group.

Conclusions

Accounting for age- related vital sign changes can more accurately detect deterioration in younger patients.

For peer review only - http://bmjopen.bmj.com/site/about/guidelines.xhtml

Strengths and limitations of this study

- We propose a data-driven early warning score using a large dataset that achieves state-of-the-art performance of clinical benchmarks.
- Our work suggests that accounting for age- related vital sign changes can more accurately detect deterioration in younger patients.
- Multi-center evaluation of early warning scores is essential to ensure that proposed models are not overfitting to the datasets used during development.
- False alarms of early warning models must be addressed by solutions that overcome the challenge of imbalanced datasets during model development.
- We assume the data to be independent and identically distributed random sets as in related work, which may limit information retrieval from rich time series data - suggesting an opportunity to build models that can recognise and learn from temporal patterns.

Introduction

Despite efforts to improve patient care, in-hospital patients still suffer unexpected adverse events such as unplanned ICU admission or cardiac arrest, which at times result in death due to late detection of serious vital-sign abnormalities and lack of timely response (1, 2). Early Warning Score (EWS) systems, also known as 'track-and-trigger' systems, assign weights to routinely collected vital-sign data based on predetermined normality ranges. They aggregate these weights to create the early warning score associated with a vital-sign observation set.

The Royal College of Physicians (RCP) launched the National Early Warning Score (NEWS) in 2012 to standardise the assessment of acutely ill adult patients across the UK, which is a modified version of the VitalPAC Early Warning Score (ViEWS) (3). NEWS assigns scores to seven physiological parameters and aggregates their scores to identify patients who are at risk of deterioration . NEWS has been shown to have superior performance to other vital signs based early warning scores (4).

Cumulative distribution functions (cdfs) determined the normality ranges of vital signs in the Centile-based Early Warning Score (CEWS) and thereafter the Manual Centile-based Early Warning Score (MCEWS) systems (5, 6). Similar to NEWS, they do not account for physiological variations across age. Previous clinical studies have investigated the pathological and physiological changes that occur with increasing age that may alter vital signs, such as for heart rate (7), blood pressure (7–11), temperature, and respiratory rate (12). The median age in the development sets for both ViEWS and MCEWS was greater than 60 years, meaning both scores were optimised to perform in an older age group. We

For peer review only - http://bmjopen.bmj.com/site/about/guidelines.xhtml

hypothesised that accounting for physiological variations across age in an EWS system, developed using a centile-based approach (5), may increase our ability to predict unanticipated ICU admission, cardiac arrest or mortality amongst hospitalised patients within 24 hours of an observation time. Differences would most likely be seen in younger age groups, as these made up a minority of the development sets of previous scores. In this study, we propose the new Age- specific Early Warning Score (ASEWS) system and describe its development and validation.

Materials and Methods

This study is reported following the TRIPOD guidance (13).

Data Source

We used a retrospective large dataset of routinely collected observations from concluded hospital admissions within the “Hospital Alerting Via Electronic Noticeboard” (HAVEN) project (REC reference: 16/SC/0264 and Confidential Advisory Group reference 08/02/1394). The database includes vital-sign measurements of adult patients, aged at least 16 years, hospitalised in any of four hospitals of the Oxford University Hospitals (OUH): John Radcliffe Hospital, Horton General Hospital, Churchill Hospital and the Nuffield Orthopaedic Hospital, between July 2013 and March 2018, and in Portsmouth Hospitals (PH) between January 2010 – March 2017. The data was collected using the System for Electronic Notification and Documentation (SEND, Sensyne Health) in OUH (14) and VitalPAC™ (System C) in PH (3).

We considered age, respiratory rate (RR), oxygen saturation (SPO_2), temperature (TEMP), systolic blood pressure (SBP), heart rate (HR), level of consciousness indicated by the “Alert, voice, pain, unresponsiveness” (AVPU) score and a binary variable indicating the provision or absence of supplemental oxygen as our model predictors. We also extracted the occurrences of mortality, unplanned ICU admission and cardiac arrest and defined our composite outcome as the time and date of the first occurring event of those events, as they are commonly used to develop EWS systems (4, 15). We separated the test populations into patients below and above the age of 45 to give sufficient separation from the median age of above 60 in previous score derivations (67.7 and 63.0 in ViEWS and MCEWS respectively (3, 6)).

For peer review only - http://bmjopen.bmj.com/site/about/guidelines.xhtml

We treated observation sets as independent rather than as grouped by patient admission. In the OUH and PH datasets, we only included complete observation sets (HR, RR, TEMP, SBP, and SPO_2) and excluded implausible physiological values. When the patient's consciousness level was assessed only using the Glasgow Coma Scale (GCS), we converted the GCS score to an AVPU score (3). If the GCS value was also missing, we assumed that the patient was 'Alert' to assign an EWS component score of 0. When the provision or absence of supplemental oxygen was missing, we assumed that supplemental oxygen was not provided to assign an EWS component score of 0.

We split the OUH dataset into an OUH development set and an OUH validation set by date (1st July 2013 to 31st July 2017 and 1st August 2017 to 31st March 2018 respectively), and validated our model using the OUH validation set and the PH validation set, with the latter consisting of concluded admissions between 1st January 2010 and 31st March 2017.

We did not apply any exclusion criteria on the OUH development set to obtain our normality ranges in an unsupervised manner across a heterogeneous population, as when MCEWS was developed (6). With interest in assessing the model performance on acutely ill patients, as in previous systems (3, 6), we applied an exclusion criteria on the OUH and PH validation sets. We excluded (a) elective and maternity admissions, (b) patients who were well enough to be discharged alive before midnight on the day of admission, (c) observation sets recorded after a patient had experienced an event, (d) and admissions with no observation sets recorded within the last 24 hours prior to an event. The flow charts are shown in Figure 1.

We summarised patient demographics, prevalence of adverse events, and the interquartile range for continuous and discrete vital signs for the development and validation sets.

Model Development

We developed our alerting thresholds by sub-setting our OUH development set for each age. At each age a, we defined its 'development subset' to include vital signs of patients aged between $a - \epsilon\, yrs$ and $a + \epsilon\, yrs$ old, where $\epsilon\ (yrs)$ is a user-defined constant. For example, when ϵ = 5, the development subset for 30-year-olds included vital signs of patients between 25 and 35 years old. We investigated

different values of ϵ ranging between 1 to 10 years and chose the value that would maximise the performance metric on the OUH development set.

In (5, 6), the alerting thresholds corresponded to pre-defined and fixed centiles for the alerting thresholds (namely 1%, 5%, 10%, 90%, 95%, & 99% centiles for double-sided distributions and 2%, 10% and 20% for single-sided distributions). In our work, we allowed a more flexible centiles selection process for the alerting thresholds of vital signs (HR, RR, TEMP, SBP, & SPO_2) at each age, which included a grid-based search approach to maximise the AUROC on the OUH development subset and ‘trial and error’. The normal ranges for AVPU and supplemental oxygen were adopted from NEWS, a score of 0 for Alert and a score of 3 otherwise (Voice, Pain, Unresponsive) and a score of 2 when supplemental oxygen was provided (5).

Performance Assessment

We evaluated the performance of the ASEWS, NEWS and MCEWS using the area under receiver-operating characteristics (AUROC) curve to predict the composite outcome of unplanned ICU admission, cardiac arrest or mortality in the OUH and PH validation sets within 24 hours of a vital-sign measurement.

We computed the AUROC and its binomial 95% confidence interval using a bootstrapping technique (n_b = 100) described in (16), for the overall validation sets across two age bands (16 - 45yrs, >= 45 yrs). The two age bands were chosen to allow assessment of performance in a younger median age group than used for NEWS and MCEWS. We also calculated the positive predictive values and plotted ‘efficiency EWS curves’ illustrating the sensitivity against the percentage of observations with a total EWS score greater than or equal to a given total EWS threshold, also known as positives or triggers (17). All analysis was performed using Python (version 3.5.5).

Patient and Public Involvement

Patients or the public were not involved in this study.

For peer review only - http://bmjopen.bmj.com/site/about/guidelines.xhtml

Results

Patient Cohort and Vitals Characteristics

In the OUH development set, there were 2,538,099 observation sets and 142,806 concluded patient admissions. Whereas in the OUH and PH validation sets, there were 581,571 observation sets corresponding to 25,407 patient admissions and 5,865,997 observation sets and 233,510 patient admissions respectively after applying the inclusion and exclusion criteria as shown in Figure 1. The proportion of female patients, mean age, and the prevalence of adverse events were similar across the development and validation sets, Table 1 and Table 2. The characteristics of the vital signs in the development and validation sets are shown in Table 3 and are generally similar across the datasets.

Fine-tuned Model Parameters

The optimal value of ϵ for the training subsets to achieve the best AUROC was 5 years. The subsequent optimised alerting thresholds of ASEWS are visualised as heatmaps in Figure 2, in addition to the alerting thresholds of NEWS and MCEWS.

Performance Evaluation

The performance of the early warning scores in predicting a composite outcome within 24 hours of its occurrence is summarised in Table 4. In the younger age group (16 – 45 years age group), ASEWS performed best in the OUH and PH validation sets (AUROC 0.820 [95% CI 0.815 – 0.824] and AUROC 0.840 [95% CI 0.839 – 0.841] respectively). In the total OUH validation set, ASEWS performed better than NEWS and MCEWS (AUROC 0.838 [95% CI 0.837-0.839), but NEWS remained superior in the PH validation set (AUROC 0.831 [95% CI 0.831 – 0.831]). The ROC curves are shown in Figure A1 (Appendix A).

In the OUH and PH validation sets, the trigger rates of ASEWS and NEWS were similar to achieve a sensitivity rate of 80%, i.e. to correctly identify 80% of observations that are within 24 hours of unplanned ICU admission, cardiac arrest or mortality. In the younger age group in the OUH validation set, the trigger rate of ASEWS was ~28.6% in comparison to a higher trigger rate of ~52.4% by NEWS. In the younger age group in the PH validation set, the trigger rate of ASEWS was ~26.5% in comparison to a higher trigger

For peer review only - http://bmjopen.bmj.com/site/about/guidelines.xhtml

rate of ~30.4% by NEWS. The trigger rate was similar for both EWS in the elder age group (>= 46 years old). The efficiency curves visualizing the trigger rates against sensitivity are shown in Figure A2 (Appendix A). Sample alerting thresholds of ASEWS are provided in Appendix B.

Discussion

Vital signs are known to vary with increasing age as shown in previous studies (8, 18). Despite known changes, the current best performing systems do not incorporate age as a predictive factor. We developed an age-specific early warning score (ASEWS) using statistical distributions of vital signs per age subset, rather than just adding age as a variable in our model.

Our new alerting thresholds improved the discrimination performance in younger patients in two independent validation sets, one being an external validation set. However, NEWS may remain a better tool for use in those above the age of 45, as it performed better in the PH dataset than ASEWS, possibly as a result of overfitting of ASEWS to the OUH dataset. Although the gains in the younger population were marginal, they are significant to highlight the potential advantage of modelling age in EWS systems.

We also show that, to achieve a sensitivity of 80% using ASEWS, which is an acceptable rate by our clinicians, the medical staff would respond to only half of the triggers generated by NEWS amongst younger patients in the OUH dataset, improving the efficiency of ward care. It also produces fewer alerts than NEWS in the PH validation set, which further emphasises generalizability in performance and clinical utility across unseen data. Our findings may most readily be used to maximise performance in more patient-specific, computer-calculated scores.

Our study has several limitations. First, we assume that the vital-sign observation sets of each patient are independent and identically distributed random sets and that there is no correlation between vital signs, which may not be the case in reality. However, this is the common methodology in studies of this type (3, 6), due to the clinical assumption that the extent of derangement is a sufficient indicator of deterioration.

The development and evaluation of EWS systems is also generally challenged by the low prevalence of adverse outcomes, which is even lower for the younger patients. Our solution to limited data is to group patients per age subsets. The severe class imbalance leads to low positive predictive values

For peer review only - http://bmjopen.bmj.com/site/about/guidelines.xhtml

(Table A1, Appendix A), and hence high false alarm rates, which is a common limitation of existing EWS systems across various patient cohorts (6, 19). Nevertheless, NEWS is still endorsed by the RCP and deployed in various clinical settings to assess acutely ill adult patients across the UK.

Overall, our work shows that using different thresholds for vital signs depending on a patient's age does improve overall performance, especially for younger patients. This motivates further analysis to maximise the benefits of incorporating age in existing EWS systems.

Conclusion

Our study suggests that incorporating age-specific centiles in the design of an early warning score system can improve performance and clinical utility for young patients in comparison to the best current systems.

References

1. Hodgetts T, Kenward G, Vlackonikolis I, et al.: Incidence, location and reasons for avoidable in-hospital cardiac arrest ina district general hospital. *Resucitation* 2002; 54:115–123
2. Bristow PJ, Daffurn K, Jacques T, et al.: Antecedents to hospital deaths. *Intern Med J* 2003; 31:343–348
3. Prytherch DR, Smith GB, Schmidt PE, et al.: ViEWS-Towards a national early warning score for detecting adult inpatient deterioration. *Resuscitation* 2010; 81:932–937
4. Smith GB, Prytherch DR, Meredith P, et al.: The ability of the National Early Warning Score (NEWS) to discriminate patients at risk of early cardiac arrest, unanticipated intensive care unit admission, and death. *Resuscitation* 2013; 84:465–470
5. Tarassenko L, Clifton DA, Pinsky MR, et al.: Centile-based early warning scores derived from statistical distributions of vital signs. *Resuscitation* 2011; 82:1013–1018
6. Watkinson PJ, Pimentel MAF, Clifton DA, et al.: Manual centile-based early warning scores derived from statistical distributions of observational vital-sign data. *Resuscitation* 2018; 129:55–60
7. Lakatta EG: Age-associated cardiovascular changes in health: impact on cardiovascular disease

For peer review only - http://bmjopen.bmj.com/site/about/guidelines.xhtml

in older persons. *Heart Fail Rev* 2002; 7:29–49

8. Franklin SS, Gustin W, Wong ND, et al.: Hemodynamic patterns of age-related changes in blood pressure. The Framingham Heart Study. *Circulation* 1997; 96:308–15

9. Gudmundsdottir H, Høieggen A, Stenehjem A, et al.: Hypertension in women: latest findings and clinical implications. *Ther Adv Chronic Dis* 2012; 3:137–46

10. Pinto E: Blood pressure and ageing. *Postgrad Med J* 2007; 83:109–114

11. Modena MG: Hypertension in postmenopausal women: How to approach hypertension in menopause. *High Blood Press Cardiovasc Prev* 2014; 21:201–204

12. Janssens JP, Pache JC, Nicod LP: Physiological changes in respiratory function associated with ageing. *Eur Respir J* 1999; 13:197–205

13. Moons KGM, Altman DG, Reitsma JB, et al.: Transparent Reporting of a multivariable prediction model for Individual Prognosis or Disagnosis (TRIPOD): Explanantion and Elaboration. *Ann Intern Med* 2015; 162:W1–W74

14. Wong D, Bonnici T, Knight J, et al.: SEND: a system for electronic notification and documentation of vital sign observations. *BMC Med Inform Decis Mak* 2015; 15:68

15. Churpek MM, Yuen TC, Edelson DP: Predicting clinical deterioration in the hospital: The impact of outcome selection. *Resuscitation* 2013; 84:564–568

16. Montgomery DC, George RC: Applied Statistics and Probability for Engineers. 6th ed. John Wiley & Sons; 2014.

17. Churpek MM, Yuen TC, Winslow C, et al.: Multicenter Comparison of Machine Learning Methods and Conventional Regression for Predicting Clinical Deterioration on the Wards. *Crit* 2016; 44:368–374

18. Chester JG, Rudolph JL: Vital Signs in Older Patients: Age-Related Changes. *J Am Med Dir Assoc* 2011; 12:337–343

19. Hoikka M, Silfvast T, Ala-Kokko TI: Does the prehospital National Early Warning Score predict the short-term mortality of unselected emergency patients? *Scand J Trauma Resusc Emerg Med* 2018;

For peer review only - http://bmjopen.bmj.com/site/about/guidelines.xhtml

Table 1

Comparison of demographics in terms of admissions across the development and validation sets, where percentages represent a proportion of number of admissions and SD is standard deviation.

Demographics	OUH Development Set	OUH Validation Set	PH Validation Set
Number of admissions	142,806	25,407	233,510
Number of observations	2,538,099	581,571	5,865,997
Females (%)	73,198 (51.3)	13,286 (52.3)	122910 (52.6)
Mean age (SD)	59.8 (20.3)	63.0 (21.4)	64.3 (21.1)
16 - 25 year olds (%)	10119 (7.1)	1742 (6.9)	15069 (6.5)
26 - 39 year olds (%)	18043 (12.6)	2952 (11.6)	22480 (9.6)
40 - 59 year olds (%)	36466 (25.5)	5217 (20.5)	47404 (20.3)
60 - 79 year olds (%)	50416 (35.3)	8240 (32.4)	79221(33.9)
≥ 80 year olds (%)	27762 (19.4)	7256 (28.6)	69336 (29.7)

For peer review only - http://bmjopen.bmj.com/site/about/guidelines.xhtml

Table 2

Comparison of the demographics across patients who experienced adverse events (i.e. composite outcome of unplanned ICU admission, mortality, or cardiac arrest) in terms of admissions across the development and validation sets, where percentages represent a proportion of number of admissions and SD is standard deviation.

Demographics	OUH Development Set	OUH Validation Set	PH Validation Set
Number of admissions with at least one adverse event	3052	869	9988
Number of observations	13565	3776	43,688
Females (%)	1359 (44.5)	400 (46.0)	4717 (47.2)
Mean age (SD)	70.0 (17.5)	73.8 (16.5)	76.0 (14.8)
16 - 25 year olds (%)	57 (1.9)	12 (1.4)	75 (0.8)
26 - 39 year olds (%)	172 (5.6)	35 (4.0)	215 (2.2)
40 - 59 year olds (%)	518 (17.0)	116 (13.4)	1042 (10.4)
60 - 79 year olds (%)	1189 (39.0)	302 (34.5)	3692 (37.0)
≥ 80 year olds (%)	1116 (36.6)	404 (46.5)	4964 (49.7)

For peer review only - http://bmjopen.bmj.com/site/about/guidelines.xhtml

Table 3

Comparisons of Median and Interquartile Range of Continuous Vital Signs (heart rate, systolic blood pressure, temperature, respiratory rate, and oxygen saturation) and Distribution of Discrete Variables (Alert, Voice, Pain, Unresponsive Score and provision of supplemental oxygen) across the development and validation sets. Percentages represent a proportion of total number of observations.

Variable, units	OUH Development Set	OUH Validation Set	PH Validation Set
Heart rate, beats/min (IQR)	81 (70-91)	81 (70-91)	80 (68-89)
Systolic blood pressure, mm Hg (IQR)	127 (111-140)	128 (112-142)	126 (110-140)
Temperature, ℃ (IQR)	36.4 (36.0-36.8)	36.5 (36.0-36.8)	36.7 (36.4-36.9)
Respiratory Rate, breaths/min (IQR)	17 (16-18)	17 (16-18)	17 (15-18)
Oxygen Saturation, % (IQR)	96 (95-98)	96 (95-98)	96 (95-98)
Count of Alert (%)	2485569 (97.9)	570793 (98.1)	5808889 (99.0)
Count of Voice (%)	39871 (1.6)	9285 (1.6)	41693 (0.7)
Count of Pain (%)	6614 (0.3)	1168 (0.2)	8498 (0.1)
Count of Unresponsive (%)	6045 (0.2)	325 (0.1)	6917 (0.1)
Count of provision of supplemental oxygen (%)	498672 (19.6)	93382 (16.9)	327114 (5.6)

For peer review only - http://bmjopen.bmj.com/site/about/guidelines.xhtml

Table 4

Performance of the Age- specific Early Warning Score and existing systems evaluated within 24 hours of a composite outcome compared using the (a) Area Under the Receiver operating characteristic curve (AUROC) and (b) 95% Confidence Intervals for overall validation sets, 16-45 year olds, and >= 46 year olds performed using 100 bootstraps with replacement, where number of samples per bootstrap is set to be equal to 30% of the sampled population.

EWS	OUH Validation Set		PH Validation Set	
	AUROC	(95% CI)	AUROC	(95% CI)
**Overall validation sets**				
ASEWS	0.838	0.837 – 0.839	0.827	0.827 – 0.828
NEWS	0.830	0.828 – 0.831	0.831	0.831 – 0.831
MCEWS	0.821	0.820 – 0.822	0.806	0.805 – 0.806
**16 – 45 years old**				
ASEWS	0.820	0.815 – 0.824	0.840	0.839 – 0.841
NEWS	0.763	0.758 – 0.768	0.836	0.835 – 0.838
MCEWS	0.808	0.803 – 0.812	0.833	0.831 – 0.834
**>= 46 years old**				
ASEWS	0.839	0.838 – 0.840	0.825	0.825 – 0.825
NEWS	0.836	0.835 – 0.837	0.830	0.829 – 0.830
MCEWS	0.821	0.820 – 0.823	0.803	0.803 – 0.804

Figure Legends

Figure 1.

Flow chart of the dataset extraction while applying inclusion and exclusion criteria for the (a) OUH Development and Validation sets and (b) PH Validation Set.

Figure 2.

Visualization of alerting thresholds for the Age-specific Early Warning Score (ASEWS), National Early Warning Score (NEWS) and Manual Centile-based Early Warning Score (MCEWS) for heart rate (HR), systolic blood pressure (SBP), temperature (TEMP), respiratory rate (RR), and oxygen saturation (SPO_2). Red indicates an alerting score of 3, orange a score of 2, yellow a score of 1, and green a score of 0.

For peer review only - http://bmjopen.bmj.com/site/about/guidelines.xhtml

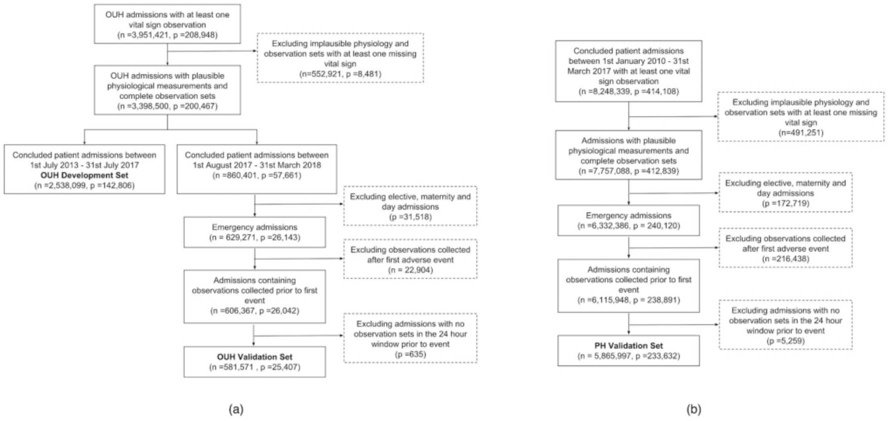

Flow chart of the dataset extraction while applying inclusion and exclusion criteria for the (a) OUH Development and Validation sets and (b) PH Validation Set.

169x72mm (300 x 300 DPI)

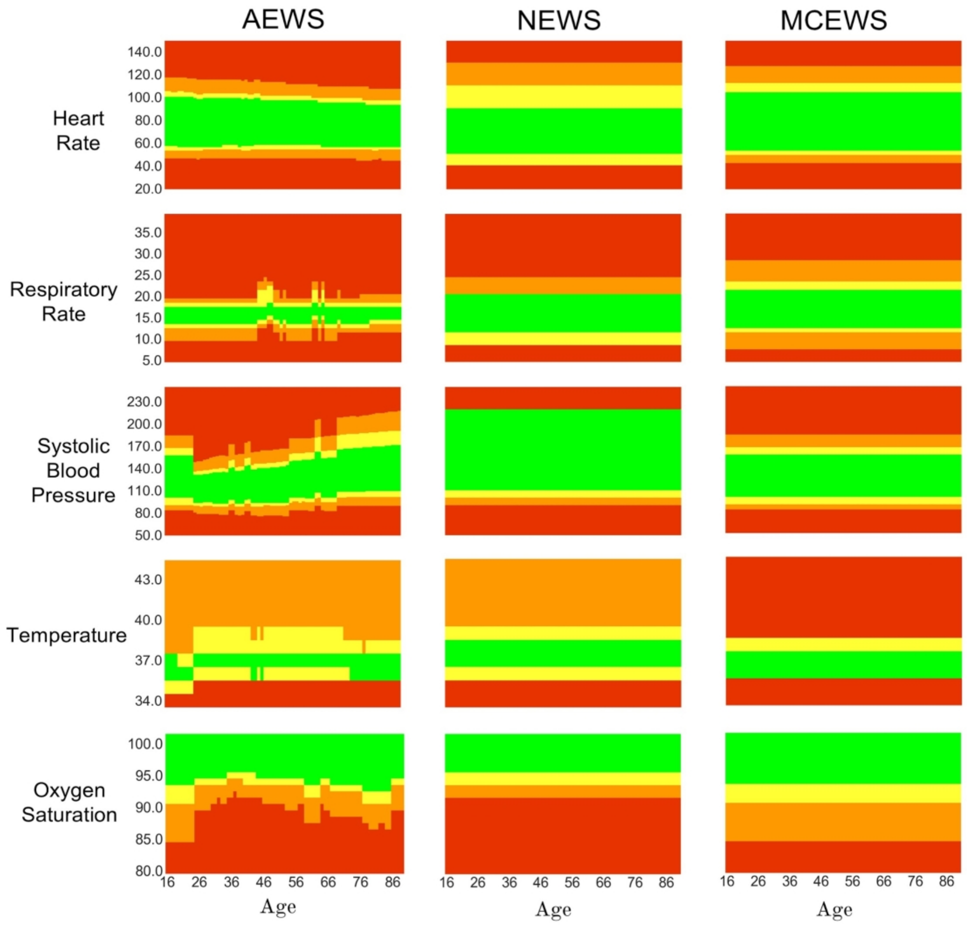

Visualization of alerting thresholds for the Age-specific Early Warning Score (ASEWS), National Early Warning Score (NEWS) and Manual Centile-based Early Warning Score (MCEWS) for heart rate (HR), systolic blood pressure (SBP), temperature (TEMP), respiratory rate (RR), and oxygen saturation (SPO2). Red indicates an alerting score of 3, orange a score of 2, yellow a score of 1, and green a score of 0.

250x246mm (300 x 300 DPI)

Appendix A

Figure A1. The receiver operating characteristics (ROC) curve evaluated on the two validation sets, Oxford University Hospitals NHS Trust denoted as 'OUH validation set' and the Portsmouth NHS Trust validation set, for the Age-specific Early Warning Score (ASEWS), Manual Centile-based Early Warning (MCEWS), and the National Early Warning Score (NEWS), for the combined outcome of mortality, cardiac arrest or unplanned ICU admission within 24 hours of vital signs observations.

OUH Validation Set **PH Validation Set**

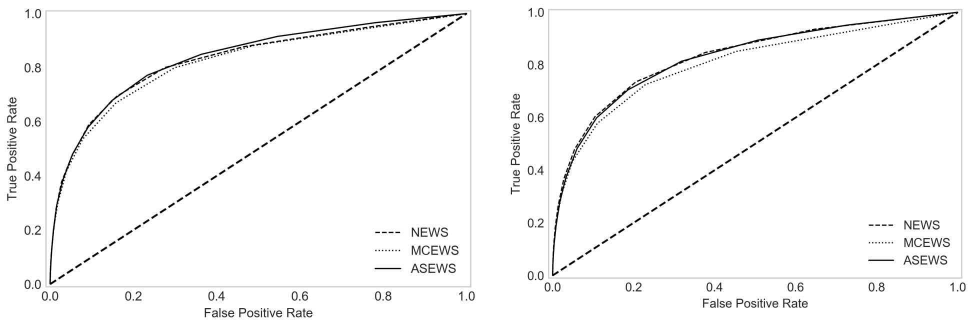

16 – 45 year olds

OUH Validation Set **PH Validation Set**

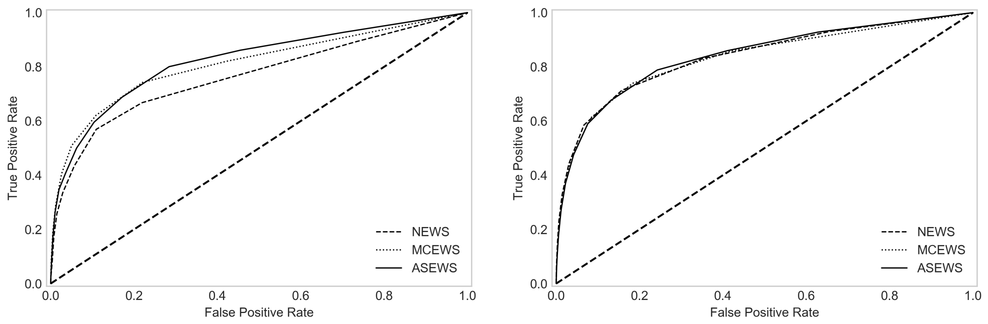

>= 46 year olds

OUH Validation Set **PH Validation Set**

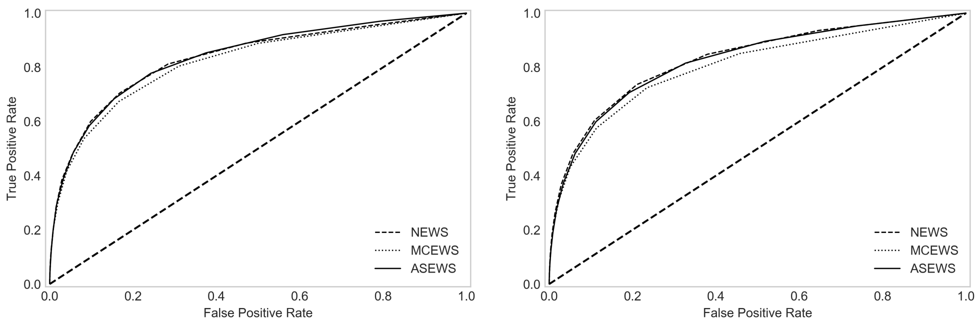

Figure A2: Efficiency EWS curves plotting sensitivity (%) (x-axis) against the percentage of observations with an EWS score greater than or equal to a given EWS threshold, denoted as positives on the y-axis, for the two best performing models, the Age-Specific Early Warning Score (ASEWS) and the National Early Warning Score (NEWS) across the overall population, 16-45 years old, and older than 45 years old in the OUH and PH validation sets. The blue lines compare the trigger rates of the EWS systems at a fixed sensitivity of 80%. The most notable difference is for the 16-45 year olds, where ASEWS produces less triggers than NEWS to achieve the same sensitivity of 80%.

(a) Overall patient population

OUH Validation Set

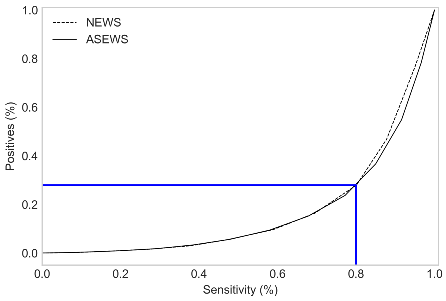

Both ASEWS and NEWS have a similar trigger rate of ~ 27.9%.

PH Validation Set

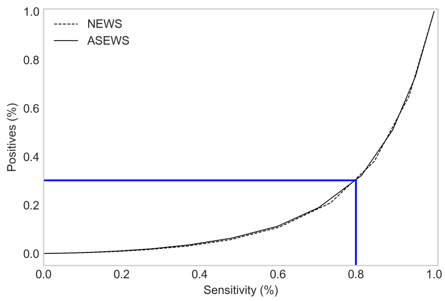

Both ASEWS and NEWS have a similar trigger rate of ~ 30.2%.

(b) 16 – 45 years old

OUH Validation Set

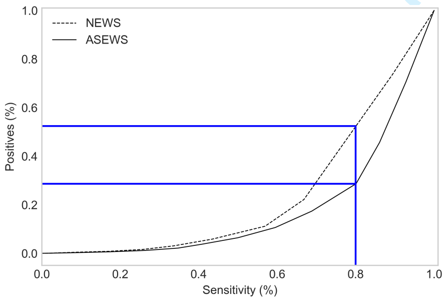

ASEWS has a trigger rate of ~ 28.6% while NEWS has a higher trigger rate of ~52.4%.

PH Validation Set

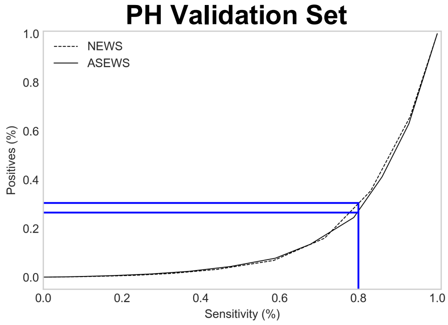

ASEWS has a trigger rate of ~ 26.5% while NEWS has a higher trigger rate of ~30.4%.

(c) >= 46 years old

OUH Validation Set

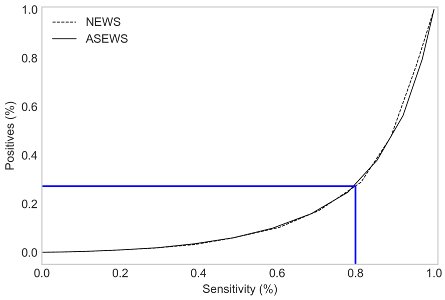

Both ASEWS and NEWS have a similar trigger rate of ~ 27.2%.

PH Validation Set

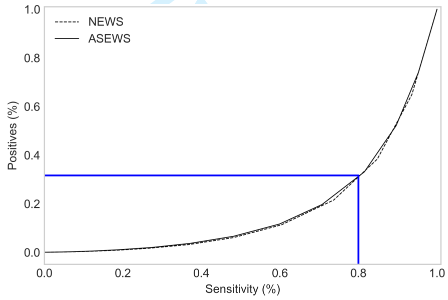

Both ASEWS and NEWS have a similar trigger rate of ~ 31.5%.

Table A1: The number of True positives (TP), False Negatives (FN), False Positives (FP), True Negatives (TN) and Positive Predictive Values (PPV) at each possible total score for the Age-specific Early Warning Score (ASEWS) and the National Early Warning Score (NEWS) as validated on the (1) OUH validation set and the (2) Portsmouth validation set for the (a) overall population, (b) 16 – 45 years old, and (c) >= 46 years old.

1. **OUH Validation Set**
   a. **Overall population**

Total EWS score	ASEWS					NEWS				
	TP	FN	FP	TN	PPV	TP	FN	FP	TN	PPV
0	3776	0	577795	0	0.01	3776	0	577795	0	0.01
1	3649	127	451477	126318	0.01	3586	190	441445	136350	0.01
2	3457	319	315372	262423	0.01	3314	462	268972	308823	0.01
3	3208	568	209897	367898	0.02	3023	753	159369	418426	0.02
4	2915	861	134765	443030	0.02	2623	1153	92603	485192	0.03
5	2561	1215	85605	492190	0.03	2225	1551	53664	524131	0.04
6	2189	1587	52549	525246	0.04	1787	1989	30078	547717	0.06
7	1814	1962	31087	546708	0.06	1422	2354	15711	562084	0.08
8	1455	2321	17725	560070	0.08	1043	2733	8131	569664	0.11
9	1107	2669	9267	568528	0.11	670	3106	3876	573919	0.15
10	751	3025	4546	573249	0.14	444	3332	1761	576034	0.2
11	470	3306	2061	575734	0.19	296	3480	811	576984	0.27
12	276	3500	870	576925	0.24	194	3582	346	577449	0.36
13	165	3611	332	577463	0.33	102	3674	139	577656	0.42
14	88	3688	130	577665	0.4	55	3721	58	577737	0.49
15	42	3734	43	577752	0.49	29	3747	14	577781	0.67
16	25	3751	12	577783	0.68	18	3758	7	577788	0.72
17	18	3758	8	577787	0.69	8	3768	1	577794	0.89
18	9	3767	3	577792	0.75	4	3772	0	577795	1.0
19	6	3770	1	577794	0.86	0	3776	0	577795	-
20	4	3772	0	577795	1.0	0	3776	0	577795	-

   b. **16-45 years old**

Total EWS score	ASEWS					NEWS				
	TP	FN	FP	TN	PPV	TP	FN	FP	TN	PPV
0	346	0	79161	0	0.0	346	0	79161	0	0.0
1	321	25	55742	23419	0.01	309	37	58067	21094	0.01
2	298	48	36011	43150	0.01	262	84	33461	45700	0.01
3	277	69	22488	56673	0.01	231	115	17359	61802	0.01
4	238	108	13548	65613	0.02	197	149	8655	70506	0.02
5	206	140	8222	70939	0.02	150	196	4464	74697	0.03
6	173	173	4914	74247	0.03	116	230	2286	76875	0.05
7	141	205	2820	76341	0.05	89	257	1159	78002	0.07
8	120	226	1568	77593	0.07	59	287	578	78583	0.09
9	91	255	825	78336	0.1	30	316	302	78859	0.09

For peer review only

10	60	286	415	78746	0.13	15	331	115	79046	0.12
11	31	315	170	78991	0.15	9	337	34	79127	0.21
12	15	331	69	79092	0.18	7	339	14	79147	0.33
13	9	337	25	79136	0.26	4	342	5	79156	0.44
14	4	342	13	79148	0.24	1	345	1	79160	0.5
15	2	344	4	79157	0.33	1	345	0	79161	1.0
16	0	346	1	79160	0.0	0	346	0	79161	-
17	0	346	0	79161	-	0	346	0	79161	-
18	0	346	0	79161	-	0	346	0	79161	-
19	0	346	0	79161	-	0	346	0	79161	-
20	0	346	0	79161	-	0	346	0	79161	-

c. >= 46 years old

Total EWS score	ASEWS					NEWS				
	TP	FN	FP	TN	PPV	TP	FN	FP	TN	PPV
0	3430	0	498634	0	0.01	3430	0	498634	0	0.01
1	3328	102	395735	102899	0.01	3277	153	383378	115256	0.01
2	3159	271	279361	219273	0.01	3052	378	235511	263123	0.01
3	2931	499	187409	311225	0.02	2792	638	142010	356624	0.02
4	2677	753	121217	377417	0.02	2426	1004	83948	414686	0.03
5	2355	1075	77383	421251	0.03	2075	1355	49200	449434	0.04
6	2016	1414	47635	450999	0.04	1671	1759	27792	470842	0.06
7	1673	1757	28267	470367	0.06	1333	2097	14552	484082	0.08
8	1335	2095	16157	482477	0.08	984	2446	7553	491081	0.12
9	1016	2414	8442	490192	0.11	640	2790	3574	495060	0.15
10	691	2739	4131	494503	0.14	429	3001	1646	496988	0.21
11	439	2991	1891	496743	0.19	287	3143	777	497857	0.27
12	261	3169	801	497833	0.25	187	3243	332	498302	0.36
13	156	3274	307	498327	0.34	98	3332	134	498500	0.42
14	84	3346	117	498517	0.42	54	3376	57	498577	0.49
15	40	3390	39	498595	0.51	28	3402	14	498620	0.67
16	25	3405	11	498623	0.69	18	3412	7	498627	0.72
17	18	3412	8	498626	0.69	8	3422	1	498633	0.89
18	9	3421	3	498631	0.75	4	3426	0	498634	1.0
19	6	3424	1	498633	0.86	0	3430	0	498634	-
20	4	3426	0	498634	1.0	0	3430	0	498634	-

For peer review only

2. **PH Validation Set**

a. **Overall population**

Total EWS score	ASEWS TP	ASEWS FN	ASEWS FP	ASEWS TN	ASEWS PPV	NEWS TP	NEWS FN	NEWS FP	NEWS TN	NEWS PPV
0	43688	0	5822309	0	0.01	43688	0	5822309	0	0.01
1	41574	2114	4239807	1582502	0.01	40884	2804	3771232	2051077	0.01
2	39067	4621	2952112	2870197	0.01	37012	6676	2193657	3628652	0.02
3	35557	8131	1852090	3970219	0.02	32127	11561	1194727	4627582	0.03
4	30810	12878	1082260	4740049	0.03	26358	17330	608462	5213847	0.04
5	26161	17527	631288	5191021	0.04	20951	22737	315354	5506955	0.06
6	21050	22638	350149	5472160	0.06	15961	27727	158894	5663415	0.09
7	16206	27482	188860	5633449	0.08	11441	32247	77263	5745046	0.13
8	11979	31709	98384	5723925	0.11	7960	35728	37033	5785276	0.18
9	8157	35531	47771	5774538	0.15	5115	38573	16233	5806076	0.24
10	5326	38362	21912	5800397	0.2	3170	40518	6838	5815471	0.32
11	3274	40414	9204	5813105	0.26	1874	41814	2682	5819627	0.41
12	1856	41832	3391	5818918	0.35	1027	42661	1040	5821269	0.5
13	995	42693	1140	5821169	0.47	515	43173	384	5821925	0.57
14	529	43159	396	5821913	0.57	239	43449	125	5822184	0.66
15	241	43447	125	5822184	0.66	92	43596	42	5822267	0.69
16	116	43572	39	5822270	0.75	33	43655	14	5822295	0.7
17	56	43632	10	5822299	0.85	11	43677	6	5822303	0.65
18	14	43674	2	5822307	0.88	3	43685	0	5822309	1.0
19	10	43678	0	5822309	1.0	1	43687	0	5822309	1.0
20	4	43684	0	5822309	1.0	1	43687	0	5822309	1.0

b. **16 – 45 years old**

Total EWS score	ASEWS TP	ASEWS FN	ASEWS FP	ASEWS TN	ASEWS PPV	NEWS TP	NEWS FN	NEWS FP	NEWS TN	NEWS PPV
0	2399	0	602429	0	0.0	2399	0	602429	0	0.0
1	2227	172	378845	223584	0.01	2233	166	396178	206251	0.01
2	2062	337	247192	355237	0.01	1993	406	212273	390156	0.01
3	1891	508	146065	456364	0.01	1712	687	95038	507391	0.02
4	1626	773	80018	522411	0.02	1403	996	39994	562435	0.03
5	1414	985	45531	556898	0.03	1060	1339	18217	584212	0.05
6	1140	1259	25143	577286	0.04	784	1615	8428	594001	0.09
7	888	1511	13450	588979	0.06	554	1845	3844	598585	0.13
8	649	1750	6997	595432	0.08	361	2038	1799	600630	0.17
9	426	1973	3339	599090	0.11	221	2178	796	601633	0.22
10	254	2145	1491	600938	0.15	146	2253	352	602077	0.29
11	147	2252	612	601817	0.19	81	2318	139	602290	0.37
12	69	2330	235	602194	0.23	39	2360	51	602378	0.43
13	38	2361	73	602356	0.34	18	2381	18	602411	0.5
14	19	2380	22	602407	0.46	8	2391	6	602423	0.57

For peer review only

15	13	2386	10	602419	0.57	1	2398	2	602427	0.33
16	4	2395	2	602427	0.67	0	2399	0	602429	-
17	2	2397	1	602428	0.67	0	2399	0	602429	-
18	0	2399	0	602429	-	0	2399	0	602429	-
19	0	2399	0	602429	-	0	2399	0	602429	-
20	0	2399	0	602429	-	0	2399	0	602429	-

c. >= 46 years old

Total EWS score	ASEWS					NEWS				
	TP	FN	FP	TN	PPV	TP	FN	FP	TN	PPV
0	41289	0	5219880	0	0.01	41289	0	5219880	0	0.01
1	39347	1942	3860962	1358918	0.01	38651	2638	3375054	1844826	0.01
2	37005	4284	2704920	2514960	0.01	35019	6270	1981384	3238496	0.02
3	33666	7623	1706025	3513855	0.02	30415	10874	1099689	4120191	0.03
4	29184	12105	1002242	4217638	0.03	24955	16334	568468	4651412	0.04
5	24747	16542	585757	4634123	0.04	19891	21398	297137	4922743	0.06
6	19910	21379	325006	4894874	0.06	15177	26112	150466	5069414	0.09
7	15318	25971	175410	5044470	0.08	10887	30402	73419	5146461	0.13
8	11330	29959	91387	5128493	0.11	7599	33690	35234	5184646	0.18
9	7731	33558	44432	5175448	0.15	4894	36395	15437	5204443	0.24
10	5072	36217	20421	5199459	0.2	3024	38265	6486	5213394	0.32
11	3127	38162	8592	5211288	0.27	1793	39496	2543	5217337	0.41
12	1787	39502	3156	5216724	0.36	988	40301	989	5218891	0.5
13	957	40332	1067	5218813	0.47	497	40792	366	5219514	0.58
14	510	40779	374	5219506	0.58	231	41058	119	5219761	0.66
15	228	41061	115	5219765	0.66	91	41198	40	5219840	0.69
16	112	41177	37	5219843	0.75	33	41256	14	5219866	0.7
17	54	41235	9	5219871	0.86	11	41278	6	5219874	0.65
18	14	41275	2	5219878	0.88	3	41286	0	5219880	1.0
19	10	41279	0	5219880	1.0	1	41288	0	5219880	1.0
20	4	41285	0	5219880	1.0	1	41288	0	5219880	1.0

For peer review only

Appendix B: Sample Alerting Thresholds of ASEWS

Age = 16

Vital Sign	3	2	1	0	1	2	3
**HR**	≥ 46	47 – 53	54 – 57	58 – 100	101 – 105	106 – 117	≤118
**RR**	≥ 9	10 – 12	13	14 – 17	18	19	≤20
**SBP**	≥ 83	84 – 90	91 – 100	101 – 157	158 – 167	168 – 184	≤185
**TEMP**	≥ 34.9		35.0 – 35.7	35.8 – 37.0	37.1 – 37.8	≤ 37.9	
**SPO_2**	≥ 84	85 – 90	91 – 93	≤ 94			
**AVPU**				A	V		P, U
**Provision of supplemental oxygen**				No		Yes	

Age = 26

Vital Sign	3	2	1	0	1	2	3
**HR**	≥ 45	46 – 53	54 – 56	57 – 98	99 – 102	103 – 115	≤116
**RR**	≥ 9	10 – 12	13	14 – 17	18	19	≤20
**SBP**	≥ 78	79 – 89	90 – 93	94 – 131	132 – 136	137 – 149	≤150
**TEMP**	≥ 35.5		35.6 – 36.0	36.1 – 37.5	37.6 – 39.5	≤ 39.6	
**SPO_2**	≥ 89	90 – 93	94	≤ 95			
**AVPU**				A	V		P, U
**Provision of supplemental oxygen**				No		Yes	

Age = 36

Vital Sign	3	2	1	0	1	2	3
**HR**	≥ 46	47 – 54	55 – 58	59 – 99	100 – 103	104 – 115	≤116
**RR**	≥ 9	10 – 12	13	14 – 17	18	19	≤20
**SBP**	≥ 83	84 – 91	92 – 97	98 – 140	141 – 150	151 – 172	≤173
**TEMP**	≥ 35.5		35.6 – 36.0	36.1 – 37.5	37.6 – 39.4	≤ 39.5	
**SPO_2**	≥ 91	92 – 94	95	≤ 96			
**AVPU**				A	V		P, U
**Provision of supplemental oxygen**				No		Yes	

Age = 46

Vital Sign	3	2	1	0	1	2	3
**HR**	≥ 46	47 – 54	55 – 58	59 – 97	98 – 101	102 – 113	≤114
**RR**	≥ 12	13	14	15 – 17	18 – 21	22 – 23	≤24
**SBP**	≥ 75	76 – 88	89 – 93	94 – 140	141 – 146	147 – 164	≤165

For peer review only

**TEMP**	≥ 35.3		35.4 – 35.9	36.0 – 37.1	37.2 – 38.3	≤ 38.4	
**SPO₂**	≥ 90	91 – 93	94	≤ 95			
**AVPU**				A	V		P, U
**Provision of supplemental oxygen**				No		Yes	

Age = 56

Vital Sign	3	2	1	0	1	2	3
**HR**	≥ 46	47 – 54	55 – 58	59 – 97	98 – 101	102 – 111	≤112
**RR**	≥ 9	10 – 12	13	14 – 17	18	19	≤20
**SBP**	≥ 83	84 – 95	96 – 100	101 – 150	151 – 160	161 – 180	≤181
**TEMP**	≥ 35.5		35.6 – 36.0	36.1 – 37.4	37.5 – 39.2	≤ 39.3	
**SPO₂**	≥ 89	90 – 93	94	≤ 95			
**AVPU**				A	V		P, U
**Provision of supplemental oxygen**				No		Yes	

Age = 66

Vital Sign	3	2	1	0	1	2	3
**HR**	≥ 46	47 – 54	55 – 56	57 – 95	96 – 99	100 – 109	≤110
**RR**	≥ 9	10 – 12	13	14 – 17	18	19	≤20
**SBP**	≥ 82	83 – 94	95 – 100	101 – 153	154 – 163	164 – 183	≤184
**TEMP**	≥ 35.5		35.6 – 36.0	36.1 – 37.4	37.5 – 39.1	≤ 39.2	
**SPO₂**	≥ 89	90 – 93	94	≤ 95			
**AVPU**				A	V		P, U
**Provision of supplemental oxygen**				No		Yes	

Age = 76

Vital Sign	3	2	1	0	1	2	3
**HR**	≥ 44	45 – 54	55 – 56	57 – 95	96 – 99	100 – 109	≤110
**RR**	≥ 11	12	13	14 – 17	18	19	≤20
**SBP**	≥ 89	90 – 101	102 – 108	109 – 167	168 – 186	187 – 209	≤210
**TEMP**	≥ 35.5		35.6 – 35.9	36.0 – 37.2	37.3 – 38.9	≤ 39.0	
**SPO₂**	≥ 88	89 – 92	93	≤ 94			
**AVPU**				A	V		P, U

For peer review only

**Provision of supplemental oxygen**				No		Yes	

Age = 86

**Vital Sign**	**3**	**2**	**1**	**0**	**1**	**2**	**3**
**HR**	≥ 44	45 – 54	55 – 56	57 – 93	94 – 97	98 – 107	≤108
**RR**	≥ 11	12 – 13	14	15 – 17	18	19 – 20	≤21
**SBP**	≥ 89	90 – 101	102 – 109	110 – 170	171 – 190	191 – 216	≤217
**TEMP**	≥ 35.4		35.5 – 35.9	36.0 – 37.1	37.2 – 38.6	≤ 38.7	
**SPO_2**	≥ 89	90 – 93	94	≤ 95			
**AVPU**				A	V		P, U
**Provision of supplemental oxygen**				No		Yes	

Age ≥ 90

**Vital Sign**	**3**	**2**	**1**	**0**	**1**	**2**	**3**
**HR**	≥ 44	45 – 54	55 – 56	57 – 93	94 – 97	98 – 107	≤108
**RR**	≥ 11	12 – 13	14	15 – 17	18	19 – 20	≤21
**SBP**	≥ 89	90 – 101	102 – 109	110 – 171	172 – 190	191 – 217	≤218
**TEMP**	≥ 35.4		35.5 – 35.9	36.0 – 37.1	37.2 – 38.6	≤ 38.7	
**SPO_2**	≥ 89	90 – 93	94	≤ 95			
**AVPU**				A	V		P, U
**Provision of supplemental oxygen**				No		Yes	

For peer review only

TRIPOD Checklist: Prediction Model Development and Validation

Section/Topic	Item		Checklist Item	Page
**Title and abstract**				
Title	1	D;V	Identify the study as developing and/or validating a multivariable prediction model, the target population, and the outcome to be predicted.	1
Abstract	2	D;V	Provide a summary of objectives, study design, setting, participants, sample size, predictors, outcome, statistical analysis, results, and conclusions.	3
**Introduction**				
Background and objectives	3a	D;V	Explain the medical context (including whether diagnostic or prognostic) and rationale for developing or validating the multivariable prediction model, including references to existing models.	4-5
	3b	D;V	Specify the objectives, including whether the study describes the development or validation of the model or both.	5
**Methods**				
Source of data	4a	D;V	Describe the study design or source of data (e.g., randomized trial, cohort, or registry data), separately for the development and validation data sets, if applicable.	5
	4b	D;V	Specify the key study dates, including start of accrual; end of accrual; and, if applicable, end of follow-up.	5
Participants	5a	D;V	Specify key elements of the study setting (e.g., primary care, secondary care, general population) including number and location of centres.	5
	5b	D;V	Describe eligibility criteria for participants.	6
	5c	D;V	Give details of treatments received, if relevant.	NA
Outcome	6a	D;V	Clearly define the outcome that is predicted by the prediction model, including how and when assessed.	5
	6b	D;V	Report any actions to blind assessment of the outcome to be predicted.	NA
Predictors	7a	D;V	Clearly define all predictors used in developing or validating the multivariable prediction model, including how and when they were measured.	5
	7b	D;V	Report any actions to blind assessment of predictors for the outcome and other predictors.	NA
Sample size	8	D;V	Explain how the study size was arrived at.	6, Fig1
Missing data	9	D;V	Describe how missing data were handled (e.g., complete-case analysis, single imputation, multiple imputation) with details of any imputation method.	5,6
Statistical analysis methods	10a	D	Describe how predictors were handled in the analyses.	6,7
	10b	D	Specify type of model, all model-building procedures (including any predictor selection), and method for internal validation.	7
	10c	V	For validation, describe how the predictions were calculated.	7
	10d	D;V	Specify all measures used to assess model performance and, if relevant, to compare multiple models.	7
	10e	V	Describe any model updating (e.g., recalibration) arising from the validation, if done.	6,7
Risk groups	11	D;V	Provide details on how risk groups were created, if done.	NA
Development vs. validation	12	V	For validation, identify any differences from the development data in setting, eligibility criteria, outcome, and predictors.	5.6
**Results**				
Participants	13a	D;V	Describe the flow of participants through the study, including the number of participants with and without the outcome and, if applicable, a summary of the follow-up time. A diagram may be helpful.	Fig 1
	13b	D;V	Describe the characteristics of the participants (basic demographics, clinical features, available predictors), including the number of participants with missing data for predictors and outcome.	Table 1,2,3
	13c	V	For validation, show a comparison with the development data of the distribution of important variables (demographics, predictors and outcome).	1,2,3
Model development	14a	D	Specify the number of participants and outcome events in each analysis.	2
	14b	D	If done, report the unadjusted association between each candidate predictor and outcome.	NA
Model specification	15a	D	Present the full prediction model to allow predictions for individuals (i.e., all regression coefficients, and model intercept or baseline survival at a given time point).	Fig 2, Appendix B
	15b	D	Explain how to the use the prediction model.	4
Model performance	16	D;V	Report performance measures (with CIs) for the prediction model.	Table 4
Model-updating	17	V	If done, report the results from any model updating (i.e., model specification, model performance).	NA
**Discussion**				
Limitations	18	D;V	Discuss any limitations of the study (such as nonrepresentative sample, few events per predictor, missing data).	9
Interpretation	19a	V	For validation, discuss the results with reference to performance in the development data, and any other validation data.	9
	19b	D;V	Give an overall interpretation of the results, considering objectives, limitations, results from similar studies, and other relevant evidence.	9
Implications	20	D;V	Discuss the potential clinical use of the model and implications for future research.	9
**Other information**				
Supplementary information	21	D;V	Provide information about the availability of supplementary resources, such as study protocol, Web calculator, and data sets.	NA
Funding	22	D;V	Give the source of funding and the role of the funders for the present study.	2

For peer review only

TRIPOD Checklist: Prediction Model Development and Validation

*Items relevant only to the development of a prediction model are denoted by D, items relating solely to a validation of a prediction model are denoted by V, and items relating to both are denoted D;V. We recommend using the TRIPOD Checklist in conjunction with the TRIPOD Explanation and Elaboration document.

For peer review only

BMJ Open

Early warning score adjusted for age to predict the composite outcome of mortality, cardiac arrest or unplanned intensive care unit admission using observational vital-sign data: a multicentre development and validation

Journal:	*BMJ Open*
Manuscript ID	bmjopen-2019-033301.R1
Article Type:	Original research
Date Submitted by the Author:	18-Sep-2019
Complete List of Authors:	Shamout, Farah; University of Oxford, Institute of Biomedical Engineering;<br>Zhu, Tingting; University of Oxford, Institute of Biomedical Engineering<br>Clifton, Lei; University of Oxford, Nuffield Department of Population Health<br>Briggs, Jim; University of Portsmouth, Centre for Healthcare Modelling and Informatics<br>Prytherch, David ; University of Portsmouth, Centre for Healthcare Modelling and Informatics<br>Meredith, Paul; Portsmouth Hospitals NHS Trust, Research & Innovation Department<br>Tarassenko, Lionel; University of Oxford, Engineering Science<br>Watkinson, Peter; Oxford University Hospitals NHS Trust, Kadoorie Centre for Critical Care research and Education<br>Clifton, David; University of Oxford, Engineering Science
**Primary Subject Heading**:	Health informatics
Secondary Subject Heading:	Intensive care
Keywords:	Health informatics < BIOTECHNOLOGY & BIOINFORMATICS, Quality in health care < HEALTH SERVICES ADMINISTRATION & MANAGEMENT, Adverse events < THERAPEUTICS

SCHOLARONE™ Manuscripts

Early warning score adjusted for age to predict the composite outcome of mortality, cardiac arrest or unplanned intensive care unit admission using observational vital-sign data: a multicentre development and validation

Farah E. Shamout (BSc)
University of Oxford
Institute of Biomedical Engineering
Old Road Campus Research Building
University of Oxford
Oxford, OX3 7DQ, UK

Tingting Zhu (DPhil)
Stipendiary Junior Research Fellowship, St Hilda's College, University of Oxford
Institute of Biomedical Engineering
Old Road Campus Research Building
University of Oxford
Oxford, OX3 7DQ, UK

Lei Clifton (PhD)
Nuffield Department of Population Health
Big Data Institute (BDI) Building
Old Road Campus Research Building
University of Oxford
Oxford, OX3 7LF, UK

Jim S. Briggs (DPhil)
Centre for Healthcare Modelling and Informatics
Buckingham Building
University of Portsmouth
Portsmouth, PO1 3HE, UK

David R. Prytherch (PhD, MIEPM, CSci)
Centre for Healthcare Modelling and Informatics
Buckingham Building
University of Portsmouth
Portsmouth, PO1 3HE, UK

Paul Meredith (PhD)
Research & Innovation
Queen Alexandra Hospital
Portsmouth Hospitals NHS Trust
PO6 3LY, UK

Lionel Tarassenko (DPhil)
Fellow of the Royal Academy of Engineering, Fellow of the Academy of Medical Sciences
Institute of Biomedical Engineering
Old Road Campus Research Building
University of Oxford
Oxford, OX3 7DQ, UK

For peer review only - http://bmjopen.bmj.com/site/about/guidelines.xhtml

Peter J. Watkinson (MD)
Nuffield Department of Clinical Neurosciences
Oxford University Hospitals NHS Trust
Oxford, OX3 9DU, UK

David A. Clifton (DPhil)
Institute of Biomedical Engineering
Old Road Campus Research Building
University of Oxford
Oxford, OX3 7DQ, UK

Address correspondence to: Farah E. Shamout, Computational Health Informatics Lab, Institute of Biomedical Engineering, University of Oxford, Oxford, OX3 7DQ, United Kingdom
Tel.: +44 (0) 7397 082634, Email: farah.shamout@balliol.ox.ac.uk

Keywords: vital signs, early warning scores, age factors, quality improvement, multicentre study, patient outcome assessment
Word Count=2,278
Abstract Word Count=300
Number of references=19
Figures=2
Tables=4
Supplementary Digital Content=2

Funding Statement

The work of FES is funded by the Rhodes Trust. PW is supported by the NIHR Biomedical Research Centre, Oxford. This publication presents independent research commissioned by the Health Innovation Challenge Fund (HICF-R9-524; WT-103703/Z/14/Z), a parallel funding partnership between the Department of Health & Social Care and Wellcome Trust. The views expressed in this publication are those of the author(s) and not necessarily those of the Department of Health & Social Care or Wellcome Trust.

Data Availability Statement

The dataset used in this study is not publicly available.

Acknowledgements

For dataset curation and extraction, we would like to thank Dr. Marco Pimentel and Dr. Oliver Redfern.

Author Contributions

Farah Shamout developed the analysis plan and undertook the data analysis and the writing of the paper. Lei Clifton guided the statistical analysis of the results. Jim Briggs, David Prytherch, & Paul Meredith collected the Portsmouth dataset and provided advice on its analysis. Peter Watkinson guided the analysis and made substantial improvements to the paper. Tingting Zhu, David Clifton and Lionel Tarassenko supervised the study and contributed to the data analysis plan.

Conflict of Interest

Lionel Tarassenko and Peter Watkinson co-developed the System for Electronic Notification and Documentation (SEND), for which Sensyne Health (SH) has purchased a sole license. SH has a research agreement with the University of Oxford and royalty agreements with Oxford University Hospitals NHS Trust and the University of Oxford. SH paid Lionel Tarassenko consultancy fees as a member of its Strategic Advisory Board. Peter Watkinson is the Chief Medical Officer and holds shares in the company. His department has received funding from SH. David Clifton is the Research Director at SH. David Prytherch was an employee of Portsmouth NHS Trust until July 2016 and he assisted the Royal College of Physicians of London in the analysis of data validating NEWS.

For peer review only - http://bmjopen.bmj.com/site/about/guidelines.xhtml

Abstract

Objectives

Early Warning Scores (EWS) alerting for in-hospital deterioration are commonly developed using routinely collected vital-sign data from the whole in-hospital population. As these in-hospital populations are dominated by those over the age of 45 years, resultant scores may perform less well in younger age groups. We developed and validated an Age-specific Early Warning Score (ASEWS) derived from statistical distributions of vital signs.

Design: Observational Cohort Study

Setting: Oxford University Hospitals (OUH) July 2013 – March 2018 and Portsmouth Hospitals (PH) NHS Trust January 2010 – March 2017 within the HAVEN database

Participants: Hospitalised patients with electronically documented vital-sign observations

Outcome: Composite outcome of unplanned intensive care unit (ICU) admission, mortality, and cardiac arrest.

Methods and Results:

Statistical distributions of vital signs were used to develop an age-specific early warning score to predict the composite outcome within 24 hours. The OUH development set consisted of 2,538,099 vital-sign observation sets from 142,806 admissions (mean age (std): 59.8 (20.3)). We compared the performance of ASEWS to the National Early Warning Score (NEWS) and our previous EWS (MCEWS) on an OUH validation set consisting of 581,571 observation sets from 25,407 emergency admissions (mean age (std): 63.0 (21.4)) and a PH validation set consisting of 5,865,997 observation sets from 233,632 emergency admissions (mean age (std): 64.3 (21.1)). ASEWS performed better in the 16-45 years age group in the OUH validation set (AUROC 0.820 [95% CI 0.815-0.824]) and PH validation set (AUROC 0.840 [95% CI 0.839-0.841]) than NEWS (AUROC 0.763 [95% CI 0.758-0.768] and AUROC 0.836 [95% CI 0.835 0 0.838] respectively) and MCEWS (AUROC 0.808 [95% CI 0.803-0.812] and AUROC 0.833 [95% CI 0.831 - 0.834] respectively). Differences in performance were not consistent in the elder age group.

Conclusions

Accounting for age- related vital sign changes can more accurately detect deterioration in younger patients.

For peer review only - http://bmjopen.bmj.com/site/about/guidelines.xhtml

Strengths and limitations of this study

- In comparison to existing heuristically-derived early warning scores, the alerting thresholds obtained in our study were derived using data mining and statistical methods.
- The score considers seven routinely collected vital signs, and thus it can be easily deployed in practice since it uses the same data sources as existing scores.
- Our approach accounts for age-related vital sign changes by sampling the development set using a sliding age window.
- Our model assumes that the data consists of independent and identically distributed random sets, therefore we do not account for changes in vital signs over time that may be indicative of deterioration.
- Another limitation is that the score was developed using data of emergency admissions, as in comparable scores, excluding elective admissions.

Introduction

Despite efforts to improve patient care, in-hospital patients still suffer unexpected adverse events such as unplanned ICU admission or cardiac arrest, which at times result in death due to late detection of serious vital-sign abnormalities and lack of timely response (1, 2). EWS systems, also known as 'track-and-trigger' systems, assign weights to routinely collected vital-sign data based on predetermined normality ranges. They aggregate these weights to create the early warning score associated with a vital-sign observation set.

The Royal College of Physicians (RCP) launched NEWS in 2012 to standardise the assessment of acutely ill adult patients across the UK, which is a modified version of the VitalPAC Early Warning Score (ViEWS) (3). NEWS assigns scores to seven physiological parameters and aggregates their scores to identify patients who are at risk of deterioration. NEWS has been shown to have superior performance to other vital signs based early warning scores (4).

Cumulative distribution functions (cdfs) determined the normality ranges of vital signs in the Centile-based Early Warning Score (CEWS) and thereafter the MCEWS systems (5, 6). Similar to NEWS, they do not account for physiological variations across age. Previous clinical studies have investigated the pathological and physiological changes that occur with increasing age that may alter vital signs, such as for heart rate (7), blood pressure (7–11), temperature, and respiratory rate (12). The median age in the development sets for both ViEWS and MCEWS was greater than 60 years, meaning both scores were optimised to perform in an older age group. We hypothesised that accounting for physiological variations

For peer review only - http://bmjopen.bmj.com/site/about/guidelines.xhtml

across age in an EWS system, developed using a centile-based approach (5), may increase our ability to predict unanticipated ICU admission, cardiac arrest or mortality amongst hospitalised patients within 24 hours of an observation time. Differences would most likely be seen in younger age groups, as these made up a minority of the development sets of previous scores. In this study, we propose the new ASEWS system and describe its development and validation.

Materials and Methods

This study is reported following the TRIPOD guidance (13).

Data Source

We used a retrospective large dataset of routinely collected observations from concluded hospital admissions within the "Hospital Alerting Via Electronic Noticeboard" (HAVEN) project (REC reference: 16/SC/0264 and Confidential Advisory Group reference 08/02/1394). The database includes vital-sign measurements of adult patients, aged at least 16 years, hospitalised in any of four hospitals of the OUH: John Radcliffe Hospital (large university hospital), Horton General Hospital (small district general hospital), Churchill Hospital (large university cancer centre) and the Nuffield Orthopaedic Hospital, between July 2013 and March 2018, and in a single large district general hospital PH between January 2010 – March 2017 (Further details on the hospitals can be found in Table A1, Appendix A). The data was collected using the System for Electronic Notification and Documentation (SEND, Sensyne Health) in OUH (14) and VitalPAC™ (System C) in PH (3).

We considered age, respiratory rate (RR), oxygen saturation (SPO_2), temperature (TEMP), systolic blood pressure (SBP), heart rate (HR), level of consciousness indicated by the "Alert, voice, pain, unresponsiveness" (AVPU) score and a binary variable indicating the provision or absence of supplemental oxygen as our model predictors. We also extracted the occurrences of mortality, unplanned ICU admission and cardiac arrest and defined our composite outcome as the time and date of the first occurring event of those events, as they are commonly used to develop EWS systems (4, 15). We separated the test

populations into patients below and above the age of 45 to give sufficient separation from the median age of above 60 in previous score derivations (67.7 and 63.0 in ViEWS and MCEWS respectively (3, 6)).

We treated observation sets as independent rather than as grouped by patient admission. In the OUH and PH datasets, we only included complete observation sets (HR, RR, TEMP, SBP, and SPO_2) and excluded implausible physiological values. When the patient's consciousness level was assessed only using the Glasgow Coma Scale (GCS), we converted the GCS score to an AVPU score (3). If the GCS value was also missing, we assumed that the patient was 'Alert' to assign an EWS component score of 0. When the provision or absence of supplemental oxygen was missing, we assumed that supplemental oxygen was not provided to assign an EWS component score of 0.

We split the OUH dataset into an OUH development set and an OUH validation set by date (1st July 2013 to 31st July 2017 and 1st August 2017 to 31st March 2018 respectively), and validated our model using the OUH validation set and the PH validation set, with the latter consisting of concluded admissions between 1st January 2010 and 31st March 2017.

We did not apply any exclusion criteria on the OUH development set to obtain our normality ranges in an unsupervised manner across a heterogeneous population, as when MCEWS was developed (6). With interest in assessing the model performance on acutely ill patients, as in previous systems (3, 6), we applied an exclusion criteria on the OUH and PH validation sets. We excluded (a) elective and maternity admissions, (b) patients who were well enough to be discharged alive before midnight on the day of admission, (c) observation sets recorded after a patient had experienced an event, (d) and admissions with no observation sets recorded within the last 24 hours prior to an event. The flow charts are shown in Figure 1.

We summarised patient demographics, prevalence of adverse events, and the interquartile range for continuous and discrete vital signs for the development and validation sets.

Model Development

We developed our alerting thresholds by sub-setting our OUH development set for each age. At each age a, we defined its 'development subset' to include vital signs of patients aged between $a - \epsilon\ yrs$ and $a + \epsilon\ yrs$ old, where $\epsilon\ (yrs)$ is a user-defined constant. For example, when ϵ = 5, the development

For peer review only - http://bmjopen.bmj.com/site/about/guidelines.xhtml

subset for 30-year-olds included vital signs of patients between 25 and 35 years old. We investigated different values of ϵ ranging between 1 to 10 years and chose the value that would maximise the performance metric on the OUH development set.

In the development of both CEWS and MCEWS (5, 6), the alerting thresholds corresponded to pre-defined and fixed centiles for the alerting thresholds (namely 1%, 5%, 10%, 90%, 95%, & 99% centiles for double-sided distributions and 2%, 10% and 20% for single-sided distributions). In our work, we allowed a more flexible centiles selection process for the alerting thresholds of vital signs (HR, RR, TEMP, SBP, & SPO_2) at each age, which included a grid-based search approach to maximise the AUROC on the OUH development subset and 'trial and error'. The normal ranges for AVPU and supplemental oxygen were adopted from NEWS, a score of 0 for Alert and a score of 3 otherwise (Voice, Pain, Unresponsive) and a score of 2 when supplemental oxygen was provided (5).

Performance Assessment

We evaluated the performance of the ASEWS, NEWS and MCEWS using the area under receiver-operating characteristics (AUROC) curve to predict the composite outcome of unplanned ICU admission, cardiac arrest or mortality in the OUH and PH validation sets within 24 hours of a vital-sign measurement. We chose an evaluation window of 24 hours as performed in previous studies (3, 6).

We computed the AUROC and its binomial 95% confidence interval using a bootstrapping technique (n_b = 100) described in (16), for the overall validation sets across two age bands (16 - 45yrs, >= 45 yrs). The two age bands were chosen to allow assessment of performance in a younger median age group than used for NEWS and MCEWS. We also calculated the positive predictive values and plotted 'efficiency EWS curves' illustrating the sensitivity against the percentage of observations with a total EWS score greater than or equal to a given total EWS threshold, also known as positives or triggers (17). All analysis was performed using Python (version 3.5.5).

Patient and Public Involvement

Patients or the public were not involved in this study.

For peer review only - http://bmjopen.bmj.com/site/about/guidelines.xhtml

Results

Patient Cohort and Vitals Characteristics

In the OUH development set, there were 2,538,099 observation sets and 142,806 concluded patient admissions. Whereas in the OUH and PH validation sets, there were 581,571 observation sets corresponding to 25,407 patient admissions and 5,865,997 observation sets and 233,632 patient admissions respectively after applying the inclusion and exclusion criteria as shown in Figure 1. The proportion of female patients, mean age, and the prevalence of adverse events were similar across the development and validation sets, Table 1 and Table 2. The characteristics of the vital signs in the development and validation sets are shown in Table 3 and are generally similar across the datasets.

Fine-tuned Model Parameters

The optimal value of ϵ for the training subsets to achieve the best AUROC was 5 years. The subsequent optimised alerting thresholds of ASEWS are visualised as heatmaps in Figure 2, in addition to the alerting thresholds of NEWS and MCEWS.

Performance Evaluation

The performance of the early warning scores in predicting a composite outcome within 24 hours of its occurrence is summarised in Table 4. In the younger age group (16 – 45 years age group), ASEWS performed best in the OUH and PH validation sets (AUROC 0.820 [95% CI 0.815 – 0.824] and AUROC 0.840 [95% CI 0.839 – 0.841] respectively). In the total OUH validation set, ASEWS performed better than NEWS and MCEWS (AUROC 0.838 [95% CI 0.837-0.839), but NEWS remained superior in the PH validation set (AUROC 0.831 [95% CI 0.831 – 0.831]). The ROC curves are shown in Figure A1 (Appendix A).

In the OUH and PH validation sets, the trigger rates of ASEWS and NEWS were similar to achieve a sensitivity rate of 80%, i.e. to correctly identify 80% of observations that are within 24 hours of unplanned ICU admission, cardiac arrest or mortality. In the younger age group in the OUH validation set, the trigger rate of ASEWS was ~28.6% in comparison to a higher trigger rate of ~52.4% by NEWS. In the younger age group in the PH validation set, the trigger rate of ASEWS was ~26.5% in comparison to a higher trigger

For peer review only - http://bmjopen.bmj.com/site/about/guidelines.xhtml

rate of ~30.4% by NEWS. The trigger rate was similar for both EWS in the elder age group (>= 46 years old). The efficiency curves visualizing the trigger rates against sensitivity are shown in Figure A2 (Appendix A). Sample alerting thresholds of ASEWS are provided in Appendix B.

Discussion

Vital signs are known to vary with increasing age as shown in previous studies (8, 18). Despite known changes, the current best performing systems do not incorporate age as a predictive factor. We developed an age-specific early warning score (ASEWS) using statistical distributions of vital signs per age subset, rather than just adding age as a variable in our model.

Our new alerting thresholds improved the discrimination performance in younger patients in two independent validation sets, one being an external validation set. However, NEWS may remain a better tool for use in those above the age of 45, as it performed better in the PH dataset than ASEWS, possibly as a result of overfitting of ASEWS to the OUH dataset. Although the gains in the younger population were marginal, they are significant to highlight the potential advantage of modelling age in EWS systems.

We also show that, to achieve a sensitivity of 80% using ASEWS, which is an acceptable rate by our clinicians, the medical staff would respond to only half of the triggers generated by NEWS amongst younger patients in the OUH dataset, improving the efficiency of ward care. It also produces fewer alerts than NEWS in the PH validation set, which further emphasises generalizability in performance and clinical utility across unseen data. Our findings may most readily be used to maximise performance in more patient-specific, computer-calculated scores.

Since the primary objective of the study is to check whether the inclusion of age has an additional value compared to existing scores, we excluded elective surgical admissions as in the development of related scores (i.e. MCEWS and NEWS). In future work, we will consider developing scores specifically for elective surgical admissions because the incidence of events across such a population is low.

Our study has several limitations. First, we assume that the vital-sign observation sets of each patient are independent and identically distributed random sets and that there is no correlation between vital signs, which may not be the case in reality. However, this is the common methodology in studies of

For peer review only - http://bmjopen.bmj.com/site/about/guidelines.xhtml

this type (3, 6), due to the clinical assumption that the extent of derangement is a sufficient indicator of deterioration.

The development and evaluation of EWS systems is also generally challenged by the low prevalence of adverse outcomes, which is even lower for the younger patients. Our solution to limited data is to group patients per age subsets. The severe class imbalance leads to low positive predictive values (Table A2, Appendix A), and hence high false alarm rates, which is a common limitation of existing EWS systems across various patient cohorts (6, 19).

Nevertheless, NEWS is still endorsed by the RCP and deployed in various clinical settings to assess acutely ill adult patients across the UK. In fact, the adoption of NEWS has been shown to be associated with lower cardiac arrests, but no associations with mortality were found (20). Involving age in such scores may be useful to enhance system efficiency and outcomes in practice.

Overall, our work shows that using different thresholds for vital signs depending on a patient's age does improve overall performance, especially for younger patients. This motivates further analysis to maximise the benefits of incorporating age in existing EWS systems.

Conclusion

Our study suggests that incorporating age-specific centiles in the design of an early warning score system can improve performance and clinical utility for young patients in comparison to the best current systems.

References

1. Hodgetts T, Kenward G, Vlackonikolis I, et al.: Incidence, location and reasons for avoidable in-hospital cardiac arrest ina district general hospital. *Resucitation* 2002; 54:115–123
2. Bristow PJ, Daffurn K, Jacques T, et al.: Antecedents to hospital deaths. *Intern Med J* 2003; 31:343–348
3. Prytherch DR, Smith GB, Schmidt PE, et al.: ViEWS-Towards a national early warning score for detecting adult inpatient deterioration. *Resuscitation* 2010; 81:932–937
4. Smith GB, Prytherch DR, Meredith P, et al.: The ability of the National Early Warning Score

For peer review only - http://bmjopen.bmj.com/site/about/guidelines.xhtml

(NEWS) to discriminate patients at risk of early cardiac arrest, unanticipated intensive care unit admission, and death. *Resuscitation* 2013; 84:465–470

5. Tarassenko L, Clifton DA, Pinsky MR, et al.: Centile-based early warning scores derived from statistical distributions of vital signs. *Resuscitation* 2011; 82:1013–1018
6. Watkinson PJ, Pimentel MAF, Clifton DA, et al.: Manual centile-based early warning scores derived from statistical distributions of observational vital-sign data. *Resuscitation* 2018; 129:55–60
7. Lakatta EG: Age-associated cardiovascular changes in health: impact on cardiovascular disease in older persons. *Heart Fail Rev* 2002; 7:29–49
8. Franklin SS, Gustin W, Wong ND, et al.: Hemodynamic patterns of age-related changes in blood pressure. The Framingham Heart Study. *Circulation* 1997; 96:308–15
9. Gudmundsdottir H, Høieggen A, Stenehjem A, et al.: Hypertension in women: latest findings and clinical implications. *Ther Adv Chronic Dis* 2012; 3:137–46
10. Pinto E: Blood pressure and ageing. *Postgrad Med J* 2007; 83:109–114
11. Modena MG: Hypertension in postmenopausal women: How to approach hypertension in menopause. *High Blood Press Cardiovasc Prev* 2014; 21:201–204
12. Janssens JP, Pache JC, Nicod LP: Physiological changes in respiratory function associated with ageing. *Eur Respir J* 1999; 13:197–205
13. Moons KGM, Altman DG, Reitsma JB, et al.: Transparent Reporting of a multivariable prediction model for Individual Prognosis or Disagnosis (TRIPOD): Explanantion and Elaboration. *Ann Intern Med* 2015; 162:W1–W74
14. Wong D, Bonnici T, Knight J, et al.: SEND: a system for electronic notification and documentation of vital sign observations. *BMC Med Inform Decis Mak* 2015; 15:68
15. Churpek MM, Yuen TC, Edelson DP: Predicting clinical deterioration in the hospital: The impact of outcome selection. *Resuscitation* 2013; 84:564–568
16. Montgomery DC, George RC: Applied Statistics and Probability for Engineers. 6th ed. John Wiley & Sons; 2014.
17. Churpek MM, Yuen TC, Winslow C, et al.: Multicenter Comparison of Machine Learning Methods

For peer review only - http://bmjopen.bmj.com/site/about/guidelines.xhtml

and Conventional Regression for Predicting Clinical Deterioration on the Wards. *Crit* 2016; 44:368–374

18. Chester JG, Rudolph JL: Vital Signs in Older Patients: Age-Related Changes. *J Am Med Dir Assoc* 2011; 12:337–343

19. Hoikka M, Silfvast T, Ala-Kokko TI: Does the prehospital National Early Warning Score predict the short-term mortality of unselected emergency patients? *Scand J Trauma Resusc Emerg Med* 2018;

20. Hogan H, Hutchings A, Wulff J, et al.: Interventions to reduce mortality from in-hospital cardiac arrest: a mixed-methods study. *Heal Serv Deliv Res* 2019; 7:1–110

For peer review only - http://bmjopen.bmj.com/site/about/guidelines.xhtml

Table 1

Comparison of demographics in terms of admissions across the development and validation sets, where percentages represent a proportion of number of admissions and SD is standard deviation.

Demographics	OUH Development Set	OUH Validation Set	PH Validation Set
Number of admissions	142,806	25,407	233,632
Number of observations	2,538,099	581,571	5,865,997
Females (%)	73,198 (51.3)	13,286 (52.3)	122910 (52.6)
Mean age (SD)	59.8 (20.3)	63.0 (21.4)	64.3 (21.1)
16 - 25 year olds (%)	10119 (7.1)	1742 (6.9)	15069 (6.5)
26 - 39 year olds (%)	18043 (12.6)	2952 (11.6)	22480 (9.6)
40 - 59 year olds (%)	36466 (25.5)	5217 (20.5)	47404 (20.3)
60 - 79 year olds (%)	50416 (35.3)	8240 (32.4)	79221(33.9)
≥ 80 year olds (%)	27762 (19.4)	7256 (28.6)	69336 (29.7)

For peer review only - http://bmjopen.bmj.com/site/about/guidelines.xhtml

Table 2

Comparison of the demographics across patients who experienced adverse events (i.e. composite outcome of unplanned ICU admission, mortality, or cardiac arrest) in terms of admissions across the development and validation sets, where percentages represent a proportion of number of admissions and SD is standard deviation.

Demographics	OUH Development Set	OUH Validation Set	PH Validation Set
Number of admissions with at least one adverse event	3052	869	9988
Number of observations	13565	3776	43,688
Females (%)	1359 (44.5)	400 (46.0)	4717 (47.2)
Mean age (SD)	70.0 (17.5)	73.8 (16.5)	76.0 (14.8)
16 - 25 year olds (%)	57 (1.9)	12 (1.4)	75 (0.8)
26 - 39 year olds (%)	172 (5.6)	35 (4.0)	215 (2.2)
40 - 59 year olds (%)	518 (17.0)	116 (13.4)	1042 (10.4)
60 - 79 year olds (%)	1189 (39.0)	302 (34.5)	3692 (37.0)
≥ 80 year olds (%)	1116 (36.6)	404 (46.5)	4964 (49.7)

For peer review only - http://bmjopen.bmj.com/site/about/guidelines.xhtml

Table 3

Comparisons of Median and Interquartile Range of Continuous Vital Signs (heart rate, systolic blood pressure, temperature, respiratory rate, and oxygen saturation) and Distribution of Discrete Variables (Alert, Voice, Pain, Unresponsive Score and provision of supplemental oxygen) across the development and validation sets. Percentages represent a proportion of total number of observations.

Variable, units	OUH Development Set	OUH Validation Set	PH Validation Set
Heart rate, beats/min (IQR)	81 (70-91)	81 (70-91)	80 (68-89)
Systolic blood pressure, mm Hg (IQR)	127 (111-140)	128 (112-142)	126 (110-140)
Temperature, ℃ (IQR)	36.4 (36.0-36.8)	36.5 (36.0-36.8)	36.7 (36.4-36.9)
Respiratory Rate, breaths/min (IQR)	17 (16-18)	17 (16-18)	17 (15-18)
Oxygen Saturation, % (IQR)	96 (95-98)	96 (95-98)	96 (95-98)
Count of Alert (%)	2485569 (97.9)	570793 (98.1)	5808889 (99.0)
Count of Voice (%)	39871 (1.6)	9285 (1.6)	41693 (0.7)
Count of Pain (%)	6614 (0.3)	1168 (0.2)	8498 (0.1)
Count of Unresponsive (%)	6045 (0.2)	325 (0.1)	6917 (0.1)
Count of provision of supplemental oxygen (%)	498672 (19.6)	93382 (16.9)	327114 (5.6)

For peer review only - http://bmjopen.bmj.com/site/about/guidelines.xhtml

Table 4

Performance of the Age- specific Early Warning Score and existing systems evaluated within 24 hours of a composite outcome compared using the (a) Area Under the Receiver operating characteristic curve (AUROC) and (b) 95% Confidence Intervals for overall validation sets, 16-45 year olds, and >= 46 year olds performed using 100 bootstraps with replacement, where number of samples per bootstrap is set to be equal to 30% of the sampled population.

EWS	OUH Validation Set		PH Validation Set	
	AUROC	(95% CI)	AUROC	(95% CI)
**Overall validation sets**				
ASEWS	0.838	0.837 – 0.839	0.827	0.827 – 0.828
NEWS	0.830	0.828 – 0.831	0.831	0.831 – 0.831
MCEWS	0.821	0.820 – 0.822	0.806	0.805 – 0.806
**16 – 45 years old**				
ASEWS	0.820	0.815 – 0.824	0.840	0.839 – 0.841
NEWS	0.763	0.758 – 0.768	0.836	0.835 – 0.838
MCEWS	0.808	0.803 – 0.812	0.833	0.831 – 0.834
**>= 46 years old**				
ASEWS	0.839	0.838 – 0.840	0.825	0.825 – 0.825
NEWS	0.836	0.835 – 0.837	0.830	0.829 – 0.830
MCEWS	0.821	0.820 – 0.823	0.803	0.803 – 0.804

For peer review only - http://bmjopen.bmj.com/site/about/guidelines.xhtml

Figure Legends

Figure 1.

Flow chart of the dataset extraction while applying inclusion and exclusion criteria for the (a) OUH Development and Validation sets and (b) PH Validation Set.

Figure 2.

Visualization of alerting thresholds for the Age-specific Early Warning Score (ASEWS), National Early Warning Score (NEWS) and Manual Centile-based Early Warning Score (MCEWS) for heart rate (HR), systolic blood pressure (SBP), temperature (TEMP), respiratory rate (RR), and oxygen saturation (SPO_2). Red indicates an alerting score of 3, orange a score of 2, yellow a score of 1, and green a score of 0.

For peer review only - http://bmjopen.bmj.com/site/about/guidelines.xhtml

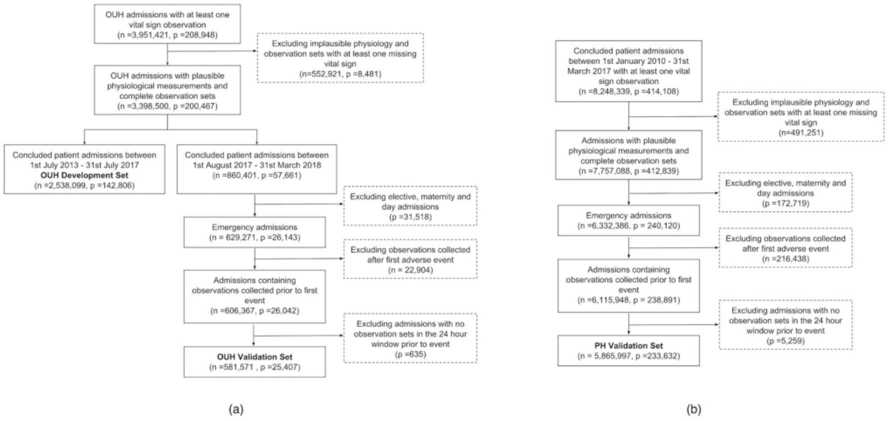

Flow chart of the dataset extraction while applying inclusion and exclusion criteria for the (a) OUH Development and Validation sets and (b) PH Validation Set.

169x72mm (300 x 300 DPI)

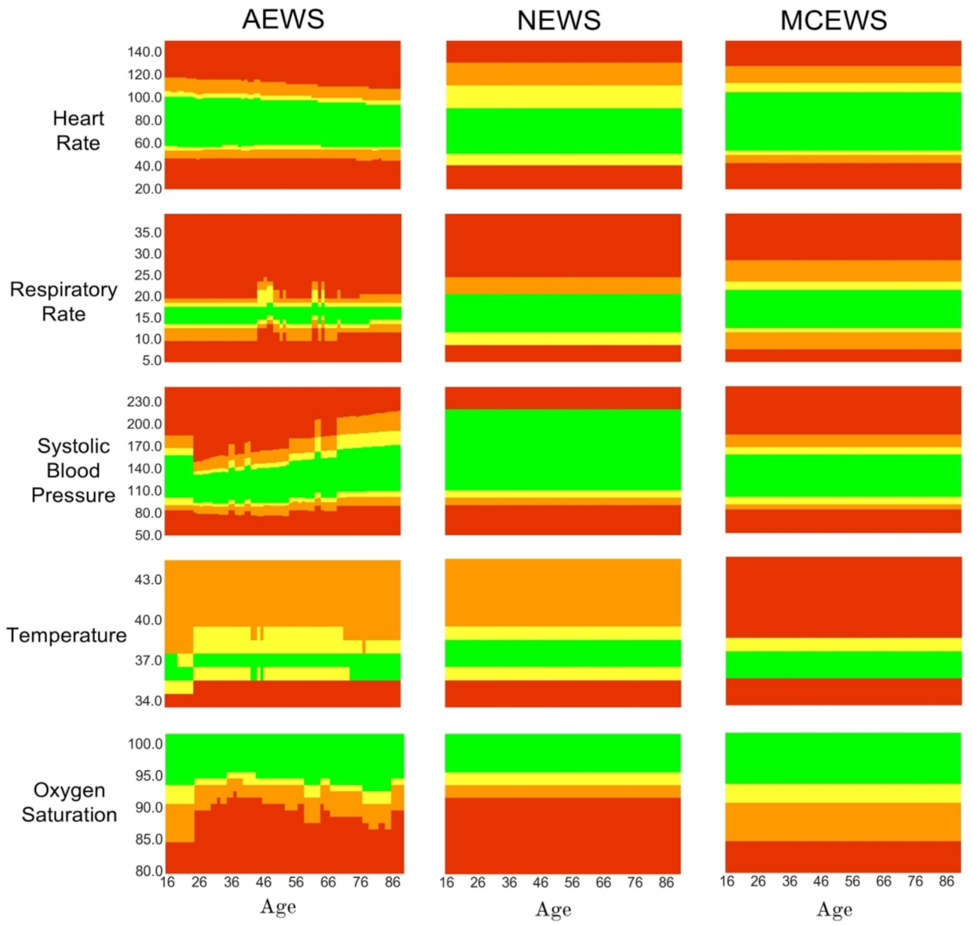

Visualization of alerting thresholds for the Age-specific Early Warning Score (ASEWS), National Early Warning Score (NEWS) and Manual Centile-based Early Warning Score (MCEWS) for heart rate (HR), systolic blood pressure (SBP), temperature (TEMP), respiratory rate (RR), and oxygen saturation (SPO2). Red indicates an alerting score of 3, orange a score of 2, yellow a score of 1, and green a score of 0.

250x246mm (300 x 300 DPI)

Appendix A

Figure A1. The receiver operating characteristics (ROC) curve evaluated on the two validation sets, Oxford University Hospitals NHS Trust denoted as ‘OUH validation set’ and the Portsmouth NHS Trust validation set, for the Age-specific Early Warning Score (ASEWS), Manual Centile-based Early Warning (MCEWS), and the National Early Warning Score (NEWS), for the combined outcome of mortality, cardiac arrest or unplanned ICU admission within 24 hours of vital signs observations.

OUH Validation Set **PH Validation Set**

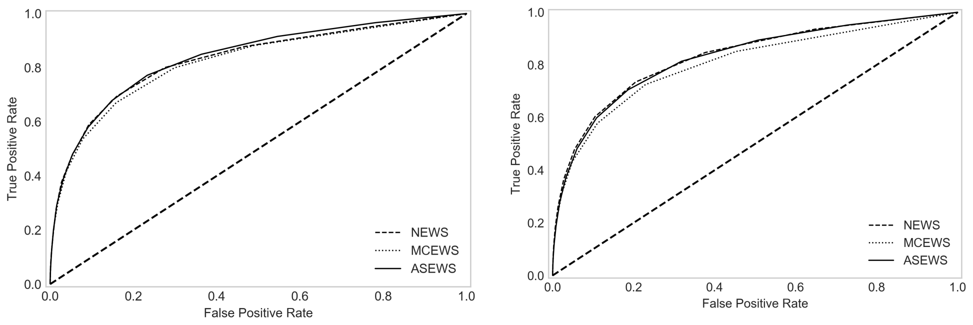

16 – 45 year olds

OUH Validation Set **PH Validation Set**

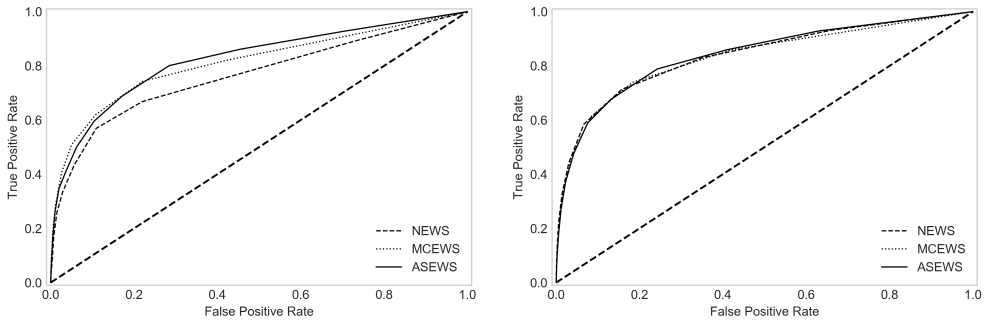

>= 46 year olds

OUH Validation Set **PH Validation Set**

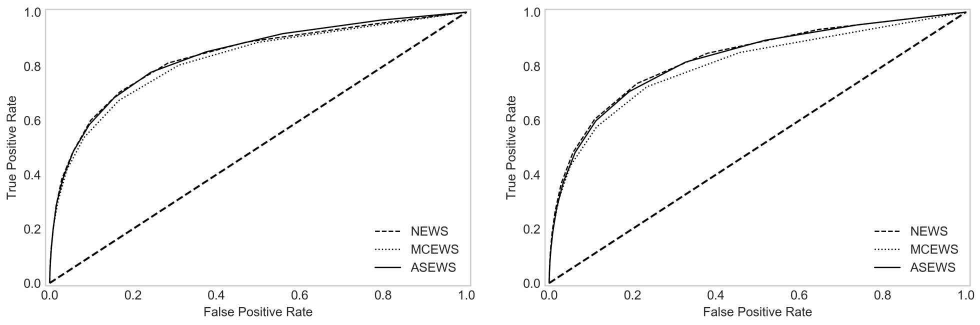

Figure A2: Efficiency EWS curves plotting sensitivity (%) (x-axis) against the percentage of observations with an EWS score greater than or equal to a given EWS threshold, denoted as positives on the y-axis, for the two best performing models, the Age-Specific Early Warning Score (ASEWS) and the National Early Warning Score (NEWS) across the overall population, 16-45 years old, and older than 45 years old in the OUH and PH validation sets. The blue lines compare the trigger rates of the EWS systems at a fixed sensitivity of 80%. The most notable difference is for the 16-45 year olds, where ASEWS produces less triggers than NEWS to achieve the same sensitivity of 80%.

(a) Overall patient population

OUH Validation Set

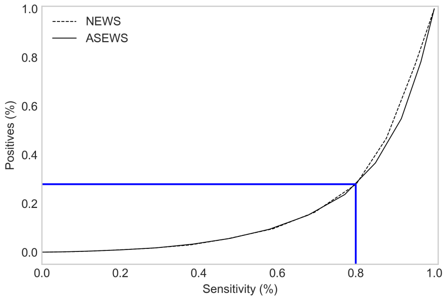

Both ASEWS and NEWS have a similar trigger rate of ~ 27.9%.

PH Validation Set

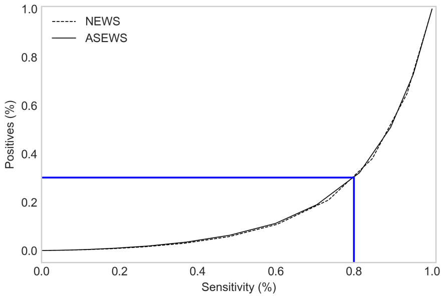

Both ASEWS and NEWS have a similar trigger rate of ~ 30.2%.

(b) 16 – 45 years old

OUH Validation Set

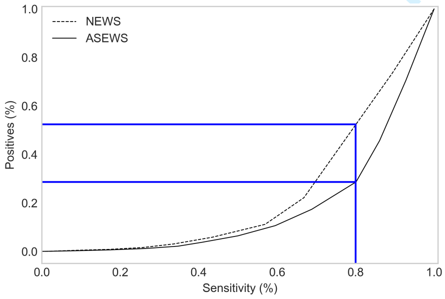

ASEWS has a trigger rate of ~ 28.6% while NEWS has a higher trigger rate of ~52.4%.

PH Validation Set

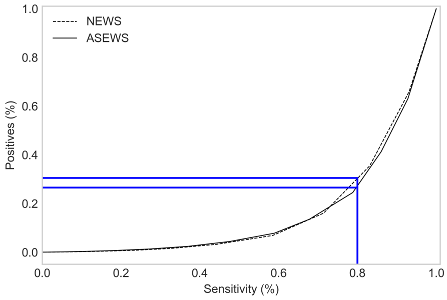

ASEWS has a trigger rate of ~ 26.5% while NEWS has a higher trigger rate of ~30.4%.

(c) >= 46 years old

OUH Validation Set

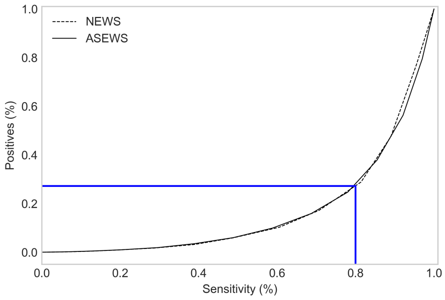

Both ASEWS and NEWS have a similar trigger rate of ~ 27.2%.

PH Validation Set

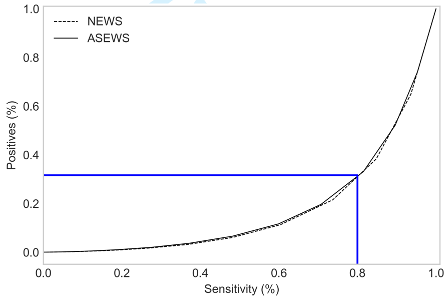

Both ASEWS and NEWS have a similar trigger rate of ~ 31.5%.

Table A1

(1) OUH Hospitals

Hospital	Size (hectares)	Catchment area	Number of beds	Percentage of attendance in 2013/2014[1]
John Radcliffe Hospital[2]	26.7	Oxfordshire	832	42.2%
Horton General Hospital[3]	9.9	Northern Oxfordshire and neighbouring communities in south Northamptonshire and south-east Warwickshire.[4]	248	11.2%
Nuffield Orthopaedic Centre[5]	8.9	Oxfordshire	160	13.4%
Churchill Hospital[6]	28.3	Oxfordshire	217	23.7%

(2) PH Hospital

Hospital	Catchment area	Number of beds	Total attendances (emergency/elective)
Queen Alexandria Hospital[7]	Portsmouth and surrounding areas of South East Hampshire	1,250	566,000

1 https://www.ouh.nhs.uk/about/publications/documents/2.TrustProfile.pdf
2 https://www.cqc.org.uk/location/RTH08/reports
3 https://www.cqc.org.uk/location/RTH05/reports
4 https://www.ouh.nhs.uk/about/publications/documents/4.MarketAssessment.pdf
5 https://www.cqc.org.uk/location/RTH03/reports
6 https://www.cqc.org.uk/sites/default/files/new_reports/AAAA0627.pdf
7 https://www.cqc.org.uk/provider/RHU/reports

For peer review only

Table A2: The number of True positives (TP), False Negatives (FN), False Positives (FP), True Negatives (TN) and Positive Predictive Values (PPV) at each possible total score for the Age-specific Early Warning Score (ASEWS) and the National Early Warning Score (NEWS) as validated on the (1) OUH validation set and the (2) Portsmouth validation set for the (a) overall population, (b) 16 – 45 years old, and (c) >= 46 years old.

1. **OUH Validation Set**

a. **Overall population**

Total EWS score	ASEWS					NEWS				
	TP	FN	FP	TN	PPV	TP	FN	FP	TN	PPV
0	3776	0	577795	0	0.01	3776	0	577795	0	0.01
1	3649	127	451477	126318	0.01	3586	190	441445	136350	0.01
2	3457	319	315372	262423	0.01	3314	462	268972	308823	0.01
3	3208	568	209897	367898	0.02	3023	753	159369	418426	0.02
4	2915	861	134765	443030	0.02	2623	1153	92603	485192	0.03
5	2561	1215	85605	492190	0.03	2225	1551	53664	524131	0.04
6	2189	1587	52549	525246	0.04	1787	1989	30078	547717	0.06
7	1814	1962	31087	546708	0.06	1422	2354	15711	562084	0.08
8	1455	2321	17725	560070	0.08	1043	2733	8131	569664	0.11
9	1107	2669	9267	568528	0.11	670	3106	3876	573919	0.15
10	751	3025	4546	573249	0.14	444	3332	1761	576034	0.2
11	470	3306	2061	575734	0.19	296	3480	811	576984	0.27
12	276	3500	870	576925	0.24	194	3582	346	577449	0.36
13	165	3611	332	577463	0.33	102	3674	139	577656	0.42
14	88	3688	130	577665	0.4	55	3721	58	577737	0.49
15	42	3734	43	577752	0.49	29	3747	14	577781	0.67
16	25	3751	12	577783	0.68	18	3758	7	577788	0.72
17	18	3758	8	577787	0.69	8	3768	1	577794	0.89
18	9	3767	3	577792	0.75	4	3772	0	577795	1.0
19	6	3770	1	577794	0.86	0	3776	0	577795	-
20	4	3772	0	577795	1.0	0	3776	0	577795	-

b. **16-45 years old**

Total EWS score	ASEWS					NEWS				
	TP	FN	FP	TN	PPV	TP	FN	FP	TN	PPV
0	346	0	79161	0	0.0	346	0	79161	0	0.0
1	321	25	55742	23419	0.01	309	37	58067	21094	0.01
2	298	48	36011	43150	0.01	262	84	33461	45700	0.01
3	277	69	22488	56673	0.01	231	115	17359	61802	0.01
4	238	108	13548	65613	0.02	197	149	8655	70506	0.02
5	206	140	8222	70939	0.02	150	196	4464	74697	0.03
6	173	173	4914	74247	0.03	116	230	2286	76875	0.05
7	141	205	2820	76341	0.05	89	257	1159	78002	0.07
8	120	226	1568	77593	0.07	59	287	578	78583	0.09
9	91	255	825	78336	0.1	30	316	302	78859	0.09

For peer review only

10	60	286	415	78746	0.13	15	331	115	79046	0.12
11	31	315	170	78991	0.15	9	337	34	79127	0.21
12	15	331	69	79092	0.18	7	339	14	79147	0.33
13	9	337	25	79136	0.26	4	342	5	79156	0.44
14	4	342	13	79148	0.24	1	345	1	79160	0.5
15	2	344	4	79157	0.33	1	345	0	79161	1.0
16	0	346	1	79160	0.0	0	346	0	79161	-
17	0	346	0	79161	-	0	346	0	79161	-
18	0	346	0	79161	-	0	346	0	79161	-
19	0	346	0	79161	-	0	346	0	79161	-
20	0	346	0	79161	-	0	346	0	79161	-

c. >= 46 years old

Total EWS score	ASEWS					NEWS				
	**TP**	**FN**	**FP**	**TN**	**PPV**	**TP**	**FN**	**FP**	**TN**	**PPV**
0	3430	0	498634	0	0.01	3430	0	498634	0	0.01
1	3328	102	395735	102899	0.01	3277	153	383378	115256	0.01
2	3159	271	279361	219273	0.01	3052	378	235511	263123	0.01
3	2931	499	187409	311225	0.02	2792	638	142010	356624	0.02
4	2677	753	121217	377417	0.02	2426	1004	83948	414686	0.03
5	2355	1075	77383	421251	0.03	2075	1355	49200	449434	0.04
6	2016	1414	47635	450999	0.04	1671	1759	27792	470842	0.06
7	1673	1757	28267	470367	0.06	1333	2097	14552	484082	0.08
8	1335	2095	16157	482477	0.08	984	2446	7553	491081	0.12
9	1016	2414	8442	490192	0.11	640	2790	3574	495060	0.15
10	691	2739	4131	494503	0.14	429	3001	1646	496988	0.21
11	439	2991	1891	496743	0.19	287	3143	777	497857	0.27
12	261	3169	801	497833	0.25	187	3243	332	498302	0.36
13	156	3274	307	498327	0.34	98	3332	134	498500	0.42
14	84	3346	117	498517	0.42	54	3376	57	498577	0.49
15	40	3390	39	498595	0.51	28	3402	14	498620	0.67
16	25	3405	11	498623	0.69	18	3412	7	498627	0.72
17	18	3412	8	498626	0.69	8	3422	1	498633	0.89
18	9	3421	3	498631	0.75	4	3426	0	498634	1.0
19	6	3424	1	498633	0.86	0	3430	0	498634	-
20	4	3426	0	498634	1.0	0	3430	0	498634	-

2. **PH Validation Set**

a. **Overall population**

Total EWS score	ASEWS TP	ASEWS FN	ASEWS FP	ASEWS TN	ASEWS PPV	NEWS TP	NEWS FN	NEWS FP	NEWS TN	NEWS PPV
0	43688	0	5822309	0	0.01	43688	0	5822309	0	0.01
1	41574	2114	4239807	1582502	0.01	40884	2804	3771232	2051077	0.01
2	39067	4621	2952112	2870197	0.01	37012	6676	2193657	3628652	0.02
3	35557	8131	1852090	3970219	0.02	32127	11561	1194727	4627582	0.03
4	30810	12878	1082260	4740049	0.03	26358	17330	608462	5213847	0.04
5	26161	17527	631288	5191021	0.04	20951	22737	315354	5506955	0.06
6	21050	22638	350149	5472160	0.06	15961	27727	158894	5663415	0.09
7	16206	27482	188860	5633449	0.08	11441	32247	77263	5745046	0.13
8	11979	31709	98384	5723925	0.11	7960	35728	37033	5785276	0.18
9	8157	35531	47771	5774538	0.15	5115	38573	16233	5806076	0.24
10	5326	38362	21912	5800397	0.2	3170	40518	6838	5815471	0.32
11	3274	40414	9204	5813105	0.26	1874	41814	2682	5819627	0.41
12	1856	41832	3391	5818918	0.35	1027	42661	1040	5821269	0.5
13	995	42693	1140	5821169	0.47	515	43173	384	5821925	0.57
14	529	43159	396	5821913	0.57	239	43449	125	5822184	0.66
15	241	43447	125	5822184	0.66	92	43596	42	5822267	0.69
16	116	43572	39	5822270	0.75	33	43655	14	5822295	0.7
17	56	43632	10	5822299	0.85	11	43677	6	5822303	0.65
18	14	43674	2	5822307	0.88	3	43685	0	5822309	1.0
19	10	43678	0	5822309	1.0	1	43687	0	5822309	1.0
20	4	43684	0	5822309	1.0	1	43687	0	5822309	1.0

b. **16 – 45 years old**

Total EWS score	ASEWS TP	ASEWS FN	ASEWS FP	ASEWS TN	ASEWS PPV	NEWS TP	NEWS FN	NEWS FP	NEWS TN	NEWS PPV
0	2399	0	602429	0	0.0	2399	0	602429	0	0.0
1	2227	172	378845	223584	0.01	2233	166	396178	206251	0.01
2	2062	337	247192	355237	0.01	1993	406	212273	390156	0.01
3	1891	508	146065	456364	0.01	1712	687	95038	507391	0.02
4	1626	773	80018	522411	0.02	1403	996	39994	562435	0.03
5	1414	985	45531	556898	0.03	1060	1339	18217	584212	0.05
6	1140	1259	25143	577286	0.04	784	1615	8428	594001	0.09
7	888	1511	13450	588979	0.06	554	1845	3844	598585	0.13
8	649	1750	6997	595432	0.08	361	2038	1799	600630	0.17
9	426	1973	3339	599090	0.11	221	2178	796	601633	0.22
10	254	2145	1491	600938	0.15	146	2253	352	602077	0.29
11	147	2252	612	601817	0.19	81	2318	139	602290	0.37
12	69	2330	235	602194	0.23	39	2360	51	602378	0.43
13	38	2361	73	602356	0.34	18	2381	18	602411	0.5
14	19	2380	22	602407	0.46	8	2391	6	602423	0.57

For peer review only

15	13	2386	10	602419	0.57	1	2398	2	602427	0.33
16	4	2395	2	602427	0.67	0	2399	0	602429	-
17	2	2397	1	602428	0.67	0	2399	0	602429	-
18	0	2399	0	602429	-	0	2399	0	602429	-
19	0	2399	0	602429	-	0	2399	0	602429	-
20	0	2399	0	602429	-	0	2399	0	602429	-

c. >= 46 years old

**Total EWS score**	**ASEWS**					**NEWS**				
	**TP**	**FN**	**FP**	**TN**	**PPV**	**TP**	**FN**	**FP**	**TN**	**PPV**
0	41289	0	5219880	0	0.01	41289	0	5219880	0	0.01
1	39347	1942	3860962	1358918	0.01	38651	2638	3375054	1844826	0.01
2	37005	4284	2704920	2514960	0.01	35019	6270	1981384	3238496	0.02
3	33666	7623	1706025	3513855	0.02	30415	10874	1099689	4120191	0.03
4	29184	12105	1002242	4217638	0.03	24955	16334	568468	4651412	0.04
5	24747	16542	585757	4634123	0.04	19891	21398	297137	4922743	0.06
6	19910	21379	325006	4894874	0.06	15177	26112	150466	5069414	0.09
7	15318	25971	175410	5044470	0.08	10887	30402	73419	5146461	0.13
8	11330	29959	91387	5128493	0.11	7599	33690	35234	5184646	0.18
9	7731	33558	44432	5175448	0.15	4894	36395	15437	5204443	0.24
10	5072	36217	20421	5199459	0.2	3024	38265	6486	5213394	0.32
11	3127	38162	8592	5211288	0.27	1793	39496	2543	5217337	0.41
12	1787	39502	3156	5216724	0.36	988	40301	989	5218891	0.5
13	957	40332	1067	5218813	0.47	497	40792	366	5219514	0.58
14	510	40779	374	5219506	0.58	231	41058	119	5219761	0.66
15	228	41061	115	5219765	0.66	91	41198	40	5219840	0.69
16	112	41177	37	5219843	0.75	33	41256	14	5219866	0.7
17	54	41235	9	5219871	0.86	11	41278	6	5219874	0.65
18	14	41275	2	5219878	0.88	3	41286	0	5219880	1.0
19	10	41279	0	5219880	1.0	1	41288	0	5219880	1.0
20	4	41285	0	5219880	1.0	1	41288	0	5219880	1.0

For peer review only

Appendix B: Sample Alerting Thresholds of ASEWS

Age = 16

Vital Sign	3	2	1	0	1	2	3
**HR**	≥ 46	47 – 53	54 – 57	58 – 100	101 – 105	106 – 117	≤118
**RR**	≥ 9	10 – 12	13	14 – 17	18	19	≤20
**SBP**	≥ 83	84 – 90	91 – 100	101 – 157	158 – 167	168 – 184	≤185
**TEMP**	≥ 34.9		35.0 – 35.7	35.8 – 37.0	37.1 – 37.8	≤ 37.9	
**SPO_2**	≥ 84	85 – 90	91 – 93	≤ 94			
**AVPU**				A	V		P, U
**Provision of supplemental oxygen**				No		Yes	

Age = 26

Vital Sign	3	2	1	0	1	2	3
**HR**	≥ 45	46 – 53	54 – 56	57 – 98	99 – 102	103 – 115	≤116
**RR**	≥ 9	10 – 12	13	14 – 17	18	19	≤20
**SBP**	≥ 78	79 – 89	90 – 93	94 – 131	132 – 136	137 – 149	≤150
**TEMP**	≥ 35.5		35.6 – 36.0	36.1 – 37.5	37.6 – 39.5	≤ 39.6	
**SPO_2**	≥ 89	90 – 93	94	≤ 95			
**AVPU**				A	V		P, U
**Provision of supplemental oxygen**				No		Yes	

Age = 36

Vital Sign	3	2	1	0	1	2	3
**HR**	≥ 46	47 – 54	55 – 58	59 – 99	100 – 103	104 – 115	≤116
**RR**	≥ 9	10 – 12	13	14 – 17	18	19	≤20
**SBP**	≥ 83	84 – 91	92 – 97	98 – 140	141 – 150	151 – 172	≤173
**TEMP**	≥ 35.5		35.6 – 36.0	36.1 – 37.5	37.6 – 39.4	≤ 39.5	
**SPO_2**	≥ 91	92 – 94	95	≤ 96			
**AVPU**				A	V		P, U
**Provision of supplemental oxygen**				No		Yes	

Age = 46

Vital Sign	3	2	1	0	1	2	3
**HR**	≥ 46	47 – 54	55 – 58	59 – 97	98 – 101	102 – 113	≤114
**RR**	≥ 12	13	14	15 – 17	18 – 21	22 – 23	≤24
**SBP**	≥ 75	76 – 88	89 – 93	94 – 140	141 – 146	147 – 164	≤165

For peer review only

For peer review only - http://bmjopen.bmj.com/site/about/guidelines.xhtml

**TEMP**	≥ 35.3		35.4 – 35.9	36.0 – 37.1	37.2 – 38.3	≤ 38.4	
**SPO_2**	≥ 90	91 – 93	94	≤ 95			
**AVPU**				A	V		P, U
**Provision of supplemental oxygen**				No		Yes	

Age = 56

**Vital Sign**	**3**	**2**	**1**	**0**	**1**	**2**	**3**
**HR**	≥ 46	47 – 54	55 – 58	59 – 97	98 – 101	102 – 111	≤112
**RR**	≥ 9	10 – 12	13	14 – 17	18	19	≤20
**SBP**	≥ 83	84 – 95	96 – 100	101 – 150	151 – 160	161 – 180	≤181
**TEMP**	≥ 35.5		35.6 – 36.0	36.1 – 37.4	37.5 – 39.2	≤ 39.3	
**SPO_2**	≥ 89	90 – 93	94	≤ 95			
**AVPU**				A	V		P, U
**Provision of supplemental oxygen**				No		Yes	

Age = 66

**Vital Sign**	**3**	**2**	**1**	**0**	**1**	**2**	**3**
**HR**	≥ 46	47 – 54	55 – 56	57 – 95	96 – 99	100 – 109	≤110
**RR**	≥ 9	10 – 12	13	14 – 17	18	19	≤20
**SBP**	≥ 82	83 – 94	95 – 100	101 – 153	154 – 163	164 – 183	≤184
**TEMP**	≥ 35.5		35.6 – 36.0	36.1 – 37.4	37.5 – 39.1	≤ 39.2	
**SPO_2**	≥ 89	90 – 93	94	≤ 95			
**AVPU**				A	V		P, U
**Provision of supplemental oxygen**				No		Yes	

Age = 76

**Vital Sign**	**3**	**2**	**1**	**0**	**1**	**2**	**3**
**HR**	≥ 44	45 – 54	55 – 56	57 – 95	96 – 99	100 – 109	≤110
**RR**	≥ 11	12	13	14 – 17	18	19	≤20
**SBP**	≥ 89	90 – 101	102 – 108	109 – 167	168 – 186	187 – 209	≤210
**TEMP**	≥ 35.5		35.6 – 35.9	36.0 – 37.2	37.3 – 38.9	≤ 39.0	
**SPO_2**	≥ 88	89 – 92	93	≤ 94			
**AVPU**				A	V		P, U

For peer review only

**Provision of supplemental oxygen**				No		Yes	

Age = 86

**Vital Sign**	**3**	**2**	**1**	**0**	**1**	**2**	**3**
**HR**	≥ 44	45 – 54	55 – 56	57 – 93	94 – 97	98 – 107	≤108
**RR**	≥ 11	12 – 13	14	15 – 17	18	19 – 20	≤21
**SBP**	≥ 89	90 – 101	102 – 109	110 – 170	171 – 190	191 – 216	≤217
**TEMP**	≥ 35.4		35.5 – 35.9	36.0 – 37.1	37.2 – 38.6	≤ 38.7	
**SPO_2**	≥ 89	90 – 93	94	≤ 95			
**AVPU**				A	V		P, U
**Provision of supplemental oxygen**				No		Yes	

Age ≥ 90

**Vital Sign**	**3**	**2**	**1**	**0**	**1**	**2**	**3**
**HR**	≥ 44	45 – 54	55 – 56	57 – 93	94 – 97	98 – 107	≤108
**RR**	≥ 11	12 – 13	14	15 – 17	18	19 – 20	≤21
**SBP**	≥ 89	90 – 101	102 – 109	110 – 171	172 – 190	191 – 217	≤218
**TEMP**	≥ 35.4		35.5 – 35.9	36.0 – 37.1	37.2 – 38.6	≤ 38.7	
**SPO_2**	≥ 89	90 – 93	94	≤ 95			
**AVPU**				A	V		P, U
**Provision of supplemental oxygen**				No		Yes	

For peer review only

TRIPOD Checklist: Prediction Model Development and Validation

Section/Topic	Item		Checklist Item	Page
**Title and abstract**				
Title	1	D;V	Identify the study as developing and/or validating a multivariable prediction model, the target population, and the outcome to be predicted.	1
Abstract	2	D;V	Provide a summary of objectives, study design, setting, participants, sample size, predictors, outcome, statistical analysis, results, and conclusions.	3
**Introduction**				
Background and objectives	3a	D;V	Explain the medical context (including whether diagnostic or prognostic) and rationale for developing or validating the multivariable prediction model, including references to existing models.	4-5
	3b	D;V	Specify the objectives, including whether the study describes the development or validation of the model or both.	5
**Methods**				
Source of data	4a	D;V	Describe the study design or source of data (e.g., randomized trial, cohort, or registry data), separately for the development and validation data sets, if applicable.	5
	4b	D;V	Specify the key study dates, including start of accrual; end of accrual; and, if applicable, end of follow-up.	5
Participants	5a	D;V	Specify key elements of the study setting (e.g., primary care, secondary care, general population) including number and location of centres.	5
	5b	D;V	Describe eligibility criteria for participants.	6
	5c	D;V	Give details of treatments received, if relevant.	NA
Outcome	6a	D;V	Clearly define the outcome that is predicted by the prediction model, including how and when assessed.	5
	6b	D;V	Report any actions to blind assessment of the outcome to be predicted.	NA
Predictors	7a	D;V	Clearly define all predictors used in developing or validating the multivariable prediction model, including how and when they were measured.	5
	7b	D;V	Report any actions to blind assessment of predictors for the outcome and other predictors.	NA
Sample size	8	D;V	Explain how the study size was arrived at.	6, Fig1
Missing data	9	D;V	Describe how missing data were handled (e.g., complete-case analysis, single imputation, multiple imputation) with details of any imputation method.	5,6
Statistical analysis methods	10a	D	Describe how predictors were handled in the analyses.	6,7
	10b	D	Specify type of model, all model-building procedures (including any predictor selection), and method for internal validation.	7
	10c	V	For validation, describe how the predictions were calculated.	7
	10d	D;V	Specify all measures used to assess model performance and, if relevant, to compare multiple models.	7
	10e	V	Describe any model updating (e.g., recalibration) arising from the validation, if done.	6,7
Risk groups	11	D;V	Provide details on how risk groups were created, if done.	NA
Development vs. validation	12	V	For validation, identify any differences from the development data in setting, eligibility criteria, outcome, and predictors.	5.6
**Results**				
Participants	13a	D;V	Describe the flow of participants through the study, including the number of participants with and without the outcome and, if applicable, a summary of the follow-up time. A diagram may be helpful.	Fig 1
	13b	D;V	Describe the characteristics of the participants (basic demographics, clinical features, available predictors), including the number of participants with missing data for predictors and outcome.	Table 1,2,3
	13c	V	For validation, show a comparison with the development data of the distribution of important variables (demographics, predictors and outcome).	1,2,3
Model development	14a	D	Specify the number of participants and outcome events in each analysis.	2
	14b	D	If done, report the unadjusted association between each candidate predictor and outcome.	NA
Model specification	15a	D	Present the full prediction model to allow predictions for individuals (i.e., all regression coefficients, and model intercept or baseline survival at a given time point).	Fig 2, Appendix B
	15b	D	Explain how to the use the prediction model.	4
Model performance	16	D;V	Report performance measures (with CIs) for the prediction model.	Table 4
Model-updating	17	V	If done, report the results from any model updating (i.e., model specification, model performance).	NA
**Discussion**				
Limitations	18	D;V	Discuss any limitations of the study (such as nonrepresentative sample, few events per predictor, missing data).	9
Interpretation	19a	V	For validation, discuss the results with reference to performance in the development data, and any other validation data.	9
	19b	D;V	Give an overall interpretation of the results, considering objectives, limitations, results from similar studies, and other relevant evidence.	9
Implications	20	D;V	Discuss the potential clinical use of the model and implications for future research.	9
**Other information**				
Supplementary information	21	D;V	Provide information about the availability of supplementary resources, such as study protocol, Web calculator, and data sets.	NA
Funding	22	D;V	Give the source of funding and the role of the funders for the present study.	2

For peer review only

TRIPOD Checklist: Prediction Model Development and Validation

*Items relevant only to the development of a prediction model are denoted by D, items relating solely to a validation of a prediction model are denoted by V, and items relating to both are denoted D;V. We recommend using the TRIPOD Checklist in conjunction with the TRIPOD Explanation and Elaboration document.

For peer review only
